# A Quantitative Study of Internal and External Interactions of Homodimeric Glucocorticoid Receptor Using Fluorescence Cross-Correlation Spectroscopy in a Live Cell

**DOI:** 10.1038/s41598-017-04499-7

**Published:** 2017-06-28

**Authors:** Manisha Tiwari, Sho Oasa, Johtaro Yamamoto, Shintaro Mikuni, Masataka Kinjo

**Affiliations:** Laboratory of Molecular Cell Dynamics, Faculty of Advanced Life Science, Hokkaido University, Sapporo, 001-0021 Japan

## Abstract

Glucocorticoid receptor (GRα) is a well-known ligand-dependent transcription-regulatory protein. The classic view is that unliganded GRα resides in the cytoplasm, relocates to the nucleus after ligand binding, and then associates with a specific DNA sequence, namely a glucocorticoid response element (GRE), to activate a specific gene as a homodimer. It is still a puzzle, however, whether GRα forms the homodimer in the cytoplasm or in the nucleus before DNA binding or after that. To quantify the homodimerization of GRα, we constructed the spectrally different fluorescent protein tagged hGRα and applied fluorescence cross-correlation spectroscopy. First, the dissociation constant (K_d_) of mCherry_2_-fused hGRα or EGFP-fused hGRα was determined *in vitro*. Then, K_d_ of wild-type hGRα was found to be 3.00 μM in the nucleus, which was higher than that *in vitro*. K_d_ of a DNA-binding-deficient mutant was 3.51 μM in the nucleus. This similarity indicated that GRα homodimerization was not necessary for DNA binding but could take place on GRE by means of GRE as a scaffold. Moreover, cytoplasmic homodimerization was also observed using GRα mutated in the nuclear localization signal. These findings support the existence of a dynamic monomer pathway and regulation of GRα function both in the cytoplasm and nucleus.

## Introduction

Understanding the interactions and dynamic properties of biomolecules in living cells is of paramount importance in life sciences. Glucocorticoid receptor α (GRα) is a natural-steroid- and synthetic-steroid-regulated transcription factor, a member of the nuclear receptor superfamily that regulates a variety of physiological functions via several mechanisms. It is widely thought that unliganded GRα is primarily located in the cytoplasm as part of a multiprotein complex with chaperones and immunophilins^1–3^. After ligand binding, GRα is translocated to the nucleus, where it works either as a homodimer that binds to positive or negative glucocorticoid response elements (GRE) located in the promoter regions of target genes, or as a monomer that cooperates with other transcription factors to induce transcription^4–7^. In addition, the homodimer of GRα can act as a repressor in association with a negative GRE, and as a monomer can tether other transcription factors such as NF-κB^8, 9^. A number of *in vitro* studies suggest that GRα homodimerizes after ligand binding^10–14^. It has been demonstrated that two molecules of the DNA-binding domain of GRα bind to a GRE in a cooperative manner, where binding of the first molecule accelerates binding of the second molecule^10, 15, 16^. It was also reported, however, that the preformed homodimer of the GRα preferentially binds to the GRE rather than sequential binding of the monomer^17–21^. It is still unclear whether binding of GRα to the GRE is followed by simple sequential or cooperative binding of the second monomer. Several recent studies have shown homodimerization of GRα *in vivo* ^17, 20, 22^. Moreover, GRα homodimerization in the cytoplasm before translocation to the nucleus has been reported^17, 20, 23^. However, it is still a matter of debate whether GRα homodimerizes in the cytoplasm or in the nucleus *in vivo* and what the function of homodimer formation in the cytoplasm is. Thus, there are still many questions about GRα function and formation. They can be answered by analyzing the affinity properties of GRα and/or formation of a complex with associated molecules in a live cell.

To find out when and where GRα homodimerizes, we used fluorescence cross-correlation spectroscopy (FCCS) to determine the binding affinity of transiently expressed enhanced green fluorescence protein (EGFP)-fused GRα, mCherry tandem dimer (mCherry_2_) protein-fused GRα, and appropriate GRα mutants, in each case in the nucleus and cytoplasm before and after addition of ligands. FCCS is a well-investigated method for determination of direct associations between spectrally different fluorescence labeled proteins in femtoliter confocal volumes^24–30^. The femtoliter confocal volume allows us to easily resolve the measurement positions in the nucleus and cytoplasm. The parameters obtained by this method are the concentrations of the labeled particles (free and bound particles) and their diffusion constants as well as the molecular sizes of their complexes^31^. FCCS has various intracellular applications, including determination of dissociation constants (K_d_) of fluorescently labeled proteins^30, 32–36^.

In our experiments here, a positive cross-correlation was obtained in wild-type (WT) GRα after addition of dexamethasone (Dex) as a synthetic ligand. Then, K_d_ values of homodimerization of full-length WT GRα and its mutants were determined and compared in living cells. Using this approach, we were able to evaluate GRα homodimerization in the cytoplasm and in the nucleus *in situ*. Our findings support the presence of a GRα homodimer in both the cytoplasm and nucleus before association with a GRE. The diffusion properties of WT GRα and mutants in the nucleus and cytoplasm in the presence and absence of Dex were also compared using a distribution of the diffusion constants.

## Results

### Analysis of hGRα homodimerization *in vitro* using FCCS

K_d_ of homodimerization of WT hGRα *in vitro* was determined by means of a single-cell measurement system combined with fluorescence correlation spectroscopy (FCS) and a microwell: the FCS-microwell system^14^. The microwell system was upgraded to FCCS (FCCS-microwell system) to determine K_d_ of the homodimerization of GRα. U2OS cells, which do not have endogenous hGRα (Figs S2A and S15A), were transiently cotransfected with a plasmid expressing WT hGRα fused to a tandem dimer of mCherry (mCherry_2_) and EGFP (Fig. S1A and B). The tandem, mCherry_2_, was used instead of monomer mCherry^30^ because of a stronger signal of relative cross amplitude (RCA) in living cells (Fig. S3). The RCA provides a relative signal of an interaction calculated by a division of the cross-correlated amplitude by one of the autocorrelation amplitudes^26, 37^. The RCA of EGFP-mCherry_2_ was less than one, because the confocal volumes between the green and red channel were incompletely overlapped^30^ and a photobleaching of fluorescent proteins may be affected. However, the fluorescent intensity was not dramatically decreased in our experiments.

**Figure 1:**
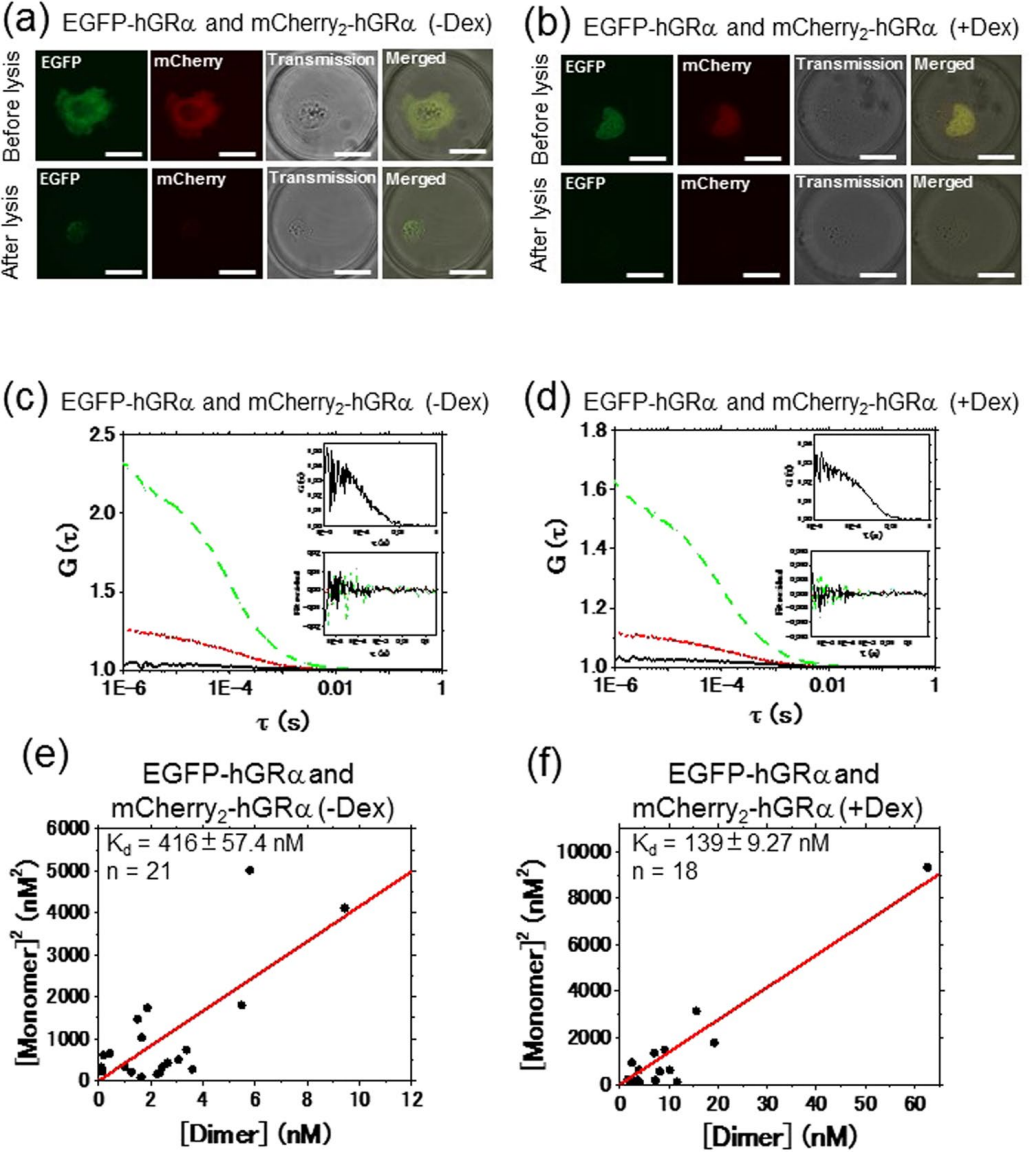
*In vitro* K_d_ analysis of EGFP-GRα and mCherry_2_-GRα using the FCCS-microwell system. Typical auto- and cross-correlation curves constructed by measurements in microwells after lysis of U2OS cells coexpressing EGFP-hGRα and mCherry_2_-hGRα in the absence or presence of Dex. The green dashed line, red dotted line, and black solid line denote the autocorrelation of the green channel [*G*_*G*_(*τ*)], autocorrelation of the red channel [*G*_*R*_(*τ*)], and cross-correlation [*G*_*C*_(*τ*)], respectively. The insets show an enlarged graph of the cross-correlation curve and fitting residuals of autocorrelation and cross-correlation curves. LSM images of U2OS cells coexpressing EGFP-hGRα and mCherry_2_-hGRα before and after cell lysis in the absence (**a**) and presence (**b**) of Dex. The scale bar is 20 μm. FCCS was performed in a microwell after cell lysis in the absence (**c**) and presence (**d**) of Dex. (**e, f**) Results of K_d_ determination using a scatter plot and linear regression. The plots represent the square of the concentration of the monomeric hGRα versus the concentration of the dimer of hGRα. The solid red line shows the linear fit. The slope indicates K_d_. (**e**) mCherry_2_-hGRα and EGFP-hGRα in the absence of Dex. (**f**) mCherry_2_-hGRα and EGFP-hGRα in the presence of Dex.

**Figure 2:**
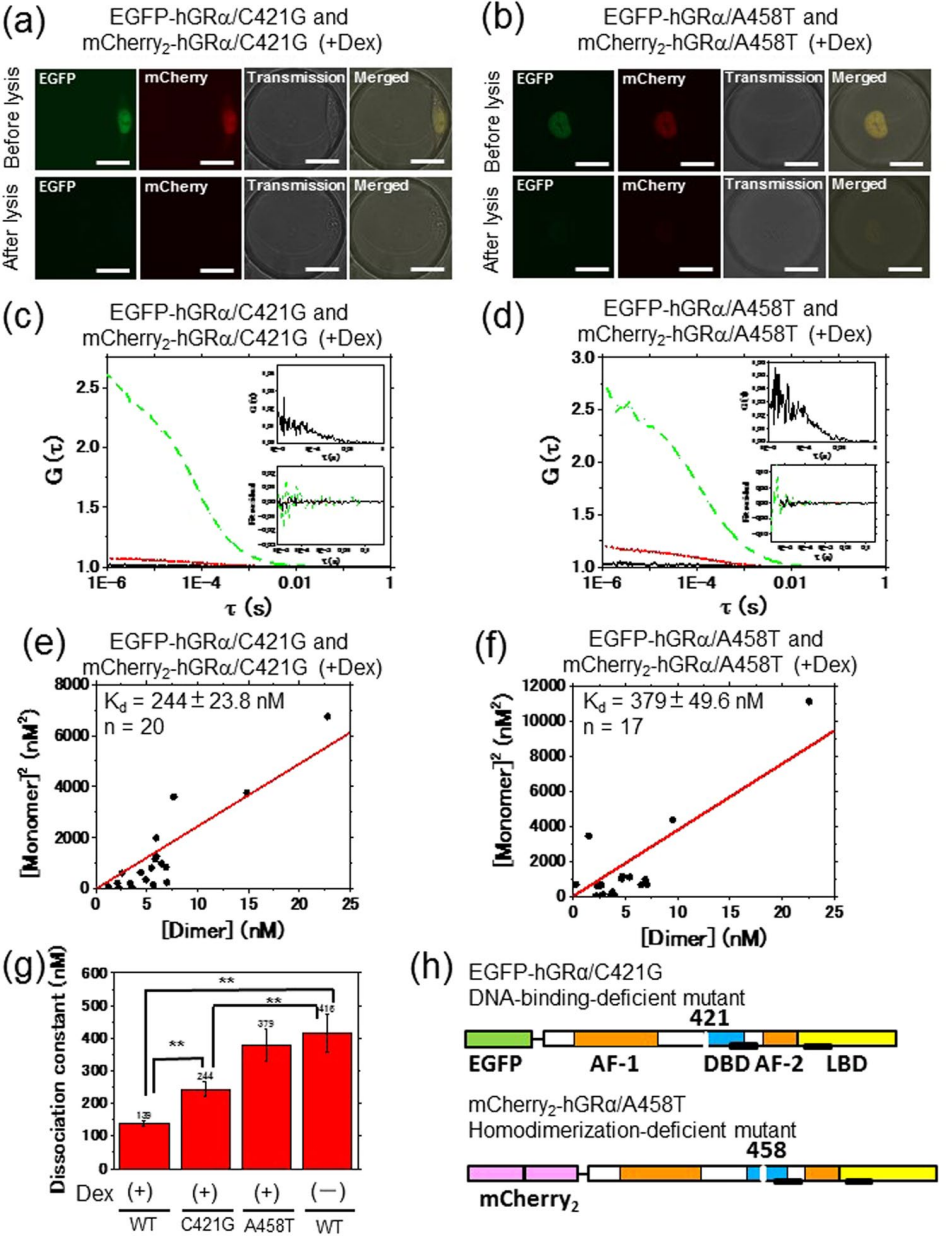
Determination of *in vitro* K_d_ of GRα mutants using the FCCS-microwell system. Typical auto- and cross-correlation curves constructed by measurements in microwells after lysis of U2OS cells coexpressing EGFP-hGRα mutants and mCherry_2_-hGRα mutants in the presence of Dex. The green dashed line, red dotted line, and black solid line denote the autocorrelation of the green channel [*G*_*G*_(*τ*)], autocorrelation of the red channel [*G*_*R*_(*τ*)], and cross-correlation [*G*_*C*_(*τ*)], respectively. The insets show an enlarged graph of the cross-correlation curve and fitting residuals of autocorrelation and cross-correlation curves. LSM images of U2OS cells coexpressing EGFP-hGRα mutants and mCherry_2_-hGRα mutants before and after cell lysis in a microwell for the C421G mutant (**a**) and A458T mutant (**b**). The scale bar is 20 μm. FCCS was performed in a microwell after cell lysis for the C421G mutant (**c**) and A458T mutant (**d**) in the presence of Dex. (**e, f**) Results of K_d_ determination using a scatter plot and linear regression. The plots represent the square of the concentration of the monomeric hGRα versus the concentration of the dimer of hGRα. The solid red line shows the linear fit. The slope indicates K_d_. (**e**) The C421G mutant in the presence of Dex. (**f**) The A458T mutant in the presence of Dex. (**g**) A summary of *in vitro* K_d_ values determined using the FCCS-microwell system. WT: wild type, C421G: the C421G mutant, A458T: the A458T mutant. Statistical analysis was based on ANOVA (**p < 0.01) (**h**) A schematic diagram of mCherry_2_- and EGFP-fused constructs of mutated hGR, C421G (DNA-binding-deficient mutant) and A458T (homodimerization-deficient mutant).

EGFP-hGRα and mCherry_2_-hGRα were localized to the cytoplasm in the absence of Dex (Fig. 1(a)) but localized to the nucleus in the presence of Dex (Fig. 1(b)). After cell lysis, the autocorrelation and cross-correlation functions were measured in the microwell (Fig. 1(c) and (d)). The RCA of the interaction between EGFP-hGRα and mCherry_2_-hGRα show similar tendencies against concentration ratio of mCherry_2_-hGRα and EGFP-hGRα (Fig. S4A), and was significantly higher than that of the negative control of EGFP and mCherry_2_, suggesting that FCCS could detect the GRα homodimerization (Fig. S4B). The concentrations of homodimeric GRα [Dimer] and monomeric GRα [Monomer] were calculated in the FCCS analysis (Supplemental information). To determine K_d_ values of GRα homodimerization, a scatter plot was generated from the square of the concentration of monomeric GRα [Monomer]^2^ and the concentration of the homodimeric GRα [Dimer], and linear regression calculation was carried out to find the best-fit line through each scatter plot by equation (15). K_d_ was calculated from the slope of the regression line^30, 32^. K_d_ of the homodimerization of WT hGRα was found to be 416 ± 57.4 and 139 ± 9.27 nM in the absence and presence of Dex, respectively (Fig. 1(e) and (f)). This K_d_ value was in good agreement with the data in our previous report determined by brightness analysis using the FCS-microwell system^14^. This consistency suggested that K_d_ values for GRα homodimerization can be determined using FCCS. Moreover, C421G (Figs 2(h) and S1C and D), a DNA-binding-deficient mutant^38^, and A458T (Figs 2(h) and S1E and F), a homodimerization-deficient mutant^39^, were analyzed using the FCCS-microwell system. The A458T mutant and C421G mutant were also localized to the nucleus in the presence of Dex (Fig. 2(a) and (b)). The autocorrelation and cross-correlation functions were then examined after cell lysis (Fig. 2(c) and (d)). K_d_ of the homodimerization of the C421G mutant and A458T mutant was found to be 244 ± 23.8 and 379 ± 49.6 nM in the presence of Dex, respectively (Fig. 2(e) and (f)).

A summary of the K_d_ values of GRα homodimerization *in vitro* is shown in Fig. 2(g). There is a significant difference between the WT in the absence and presence of Dex, suggesting that the hGRα homodimerization was induced by Dex. Moreover, K_d_ of homodimerization of the C421G mutant was significantly lower than that of the WT in the absence of Dex, and was significantly higher than K_d_ of the WT in the presence of Dex. This finding suggested that GRα homodimerization was not necessary for DNA binding but that DNA has a role of scaffolds for GRα homodimerization. The A458T mutant showed similar K_d_ value to that of the WT in the absence of Dex. These results indicated that FCCS can determine K_d_ of hGRα homodimerization.

### FCCS analysis of WT hGRα in the nucleus and cytoplasm

**Figure 3:**
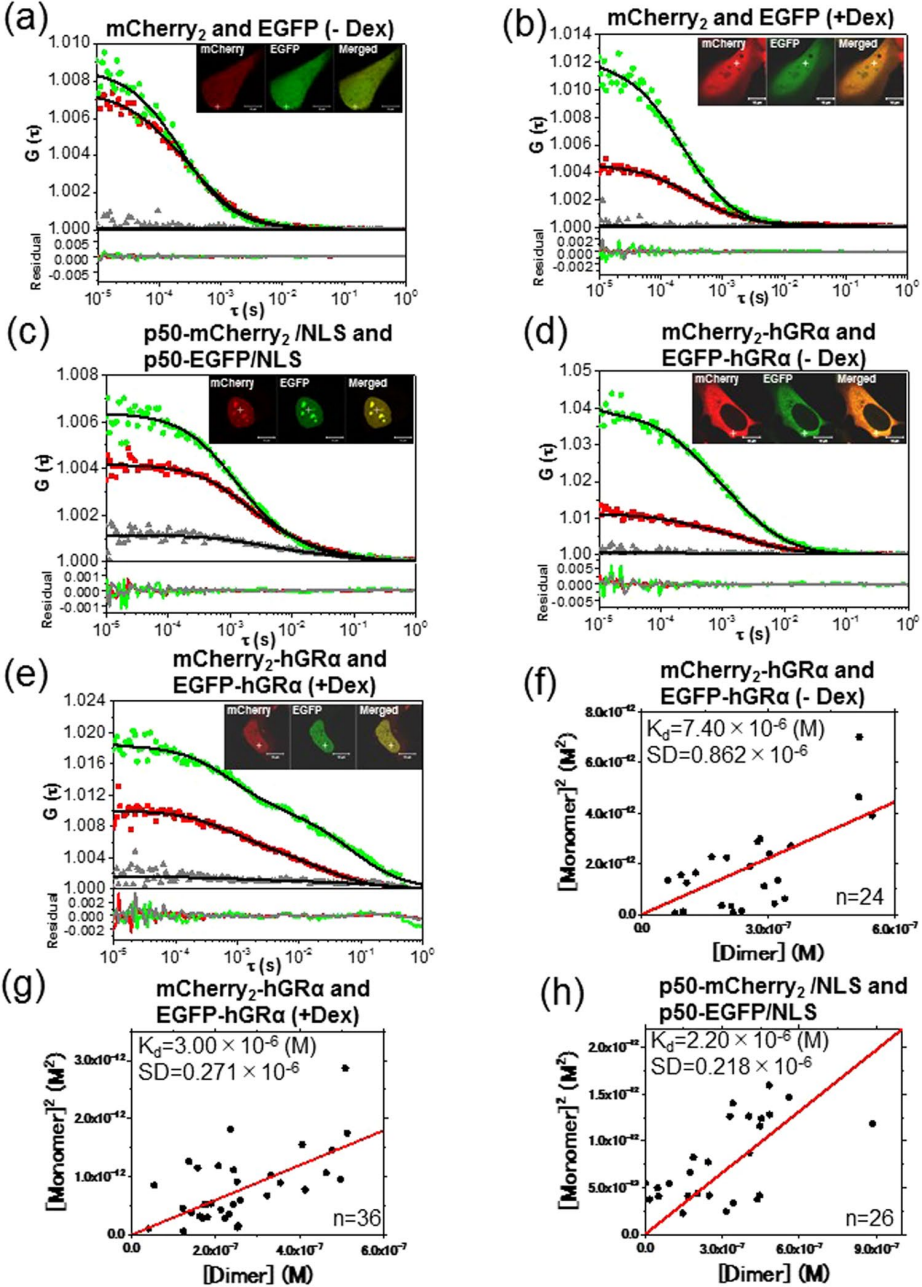
FCCS and K_d_ analysis of mCherry_2_-GRα and EGFP-GRα. Typical auto- and cross-correlation curves constructed by measurements in U2OS cells coexpressing the pairs of chimeric fusion proteins before and after addition of the ligand. The filled green diamonds, red squares, and gray triangles denote the autocorrelation of the green channel [*G*_*G*_(*τ*)], autocorrelation of the red channel [*G*_*R*_(*τ*)], and the cross-correlation curve [*G*_*C*_(*τ*)], respectively, with their fits (solid black line) and residuals. The insets show LSM images of U2OS cells coexpressing the pairs of chimeric fusion proteins. Measurement positions of FCCS are indicated by the white crosshairs. The scale bars are 10 μm. FCCS was performed in U2OS cells expressing mCherry_2_ and EGFP as a negative control (**a**) before and (**b**) after addition of Dex, showing a flat cross-correlation amplitude. (**c**) A U2OS cell coexpressing p50-mCherry_2_/NLS and p50-EGFP/NLS as a positive control. (**d**) A U2OS cell coexpressing mCherry_2_/hGRα and EGFP/hGRα in the cytoplasm before addition of Dex. (**e**) A U2OS cell coexpressing mCherry_2_ **/**hGRα and EGFP**/**hGRα in the nucleus 20 min after addition of 100 nM Dex. (**f**,**g**,**h**) Results of K_d_ determination using a scatter plot and linear regression. The plots represent the square of the concentration of the monomeric hGRα versus the concentration of the dimer of hGRα. The solid lines show the linear fit. The slope indicates K_d_. (**f**) mCherry_2_-hGRα and EGFP-hGRα before addition of Dex. (**g**) mCherry_2_-hGRα and EGFP-hGRα after addition of Dex. (**h**) p50-mCherry_2_/NLS and p50-EGFP/NLS.

To study the GRα homodimerization in living cells, FCCS was performed in living U2OS cells. The cells were transiently cotransfected with plasmid constructs expressing EGFP-hGRα and mCherry_2_-hGRα (Fig. S1A and B). The fusion proteins mCherry_2_-hGRα and EGFP-hGRα were initially localized to the cytoplasm in the absence of Dex (Fig. 3(d), inset), but after the addition of Dex, both mCherry_2_-hGRα and EGFP-hGRα were translocated to the nucleus during 20 min (Fig. 3(e), inset). As in another report^40, 41^, the transcription-regulatory activity of GRα was retained after tagging with such fluorescent proteins.

Typical autocorrelation and cross-correlation curves of FCCS conducted in the cytoplasm and nucleus are shown in Fig. 3. As a negative control, U2OS cells were cotransfected independently with mCherry_2_- and EGFP-encoding plasmids, and FCCS was carried out in the absence (Fig. 3(a)) and presence of Dex (Fig. 3(b)). The cross-correlation amplitude was not observed in either case, pointing to no unknown interaction between EGFP and mCherry_2_. As a positive control, U2OS cells were cotransfected with p50-mCherry_2_/nuclear localization signal (NLS)-encoding and p50-EGFP/NLS-encoding plasmids (Fig. S1M and N), which are coexpressed in the nucleus as the proteins of interest (Fig. 3(c), inset). The p50 protein is a subunit of NF-kB, and proteins of this family associate as a homo- (p50-p50) and heterodimer (p50-p65). Endogenous expression of p50 was not detected in U2OS cells (Figs S2B and S15B). A high cross-correlation amplitude was observed (Fig. 3(c)) between the p50-mCherry_2_/NLS and p50-EGFP/NLS in FCCS measurement. Low cross-correlation amplitude of mCherry_2_-hGRα and EGFP-hGRα was observed in the absence of Dex (Fig. 3(d)) in the cytoplasm. In contrast, a cross-correlation amplitude was observed in the presence of Dex (Fig. 3(e)) in the nucleus. For quantitative analysis, K_d_ values of the GRα homodimerization in U2OS cells were computed in the absence and presence of Dex. The RCA of the interaction between EGFP-hGRα and mCherry_2_-hGRα in the living cells show similar tendencies against concentration ratio of mCherry_2_-hGRα and EGFP-hGRα (Fig. S5A and B), and was significantly higher than that of the coexpression of EGFP and mCherry_2_, indicating that FCCS could detect the GRα homodimerization in the living cells as well as *in vitro* (Fig. S5C). K_d_ of p50-mCherry_2_/NLS and p50-EGFP/NLS in the nucleus was found to be 2.20 μM (Fig. 3(h)). This result was consistent with another report on the micromolar range of K_d_ for p50 homodimerization *in vitro* ^42^. K_d_ values of mCherry_2_-hGRα and EGFP-hGRα were found to be 7.40 μM in the absence of Dex in the cytoplasm (Fig. 3(f)) and 3.00 μM in the presence of Dex in the nucleus (Fig. 3(g)). K_d_ was significantly (p < 0.01) lower in the presence of Dex than in its absence. These quantitative results suggested that WT GRα has a tendency toward homodimerization in the presence of Dex and toward monomerization in the absence of Dex.

**Figure 4:**
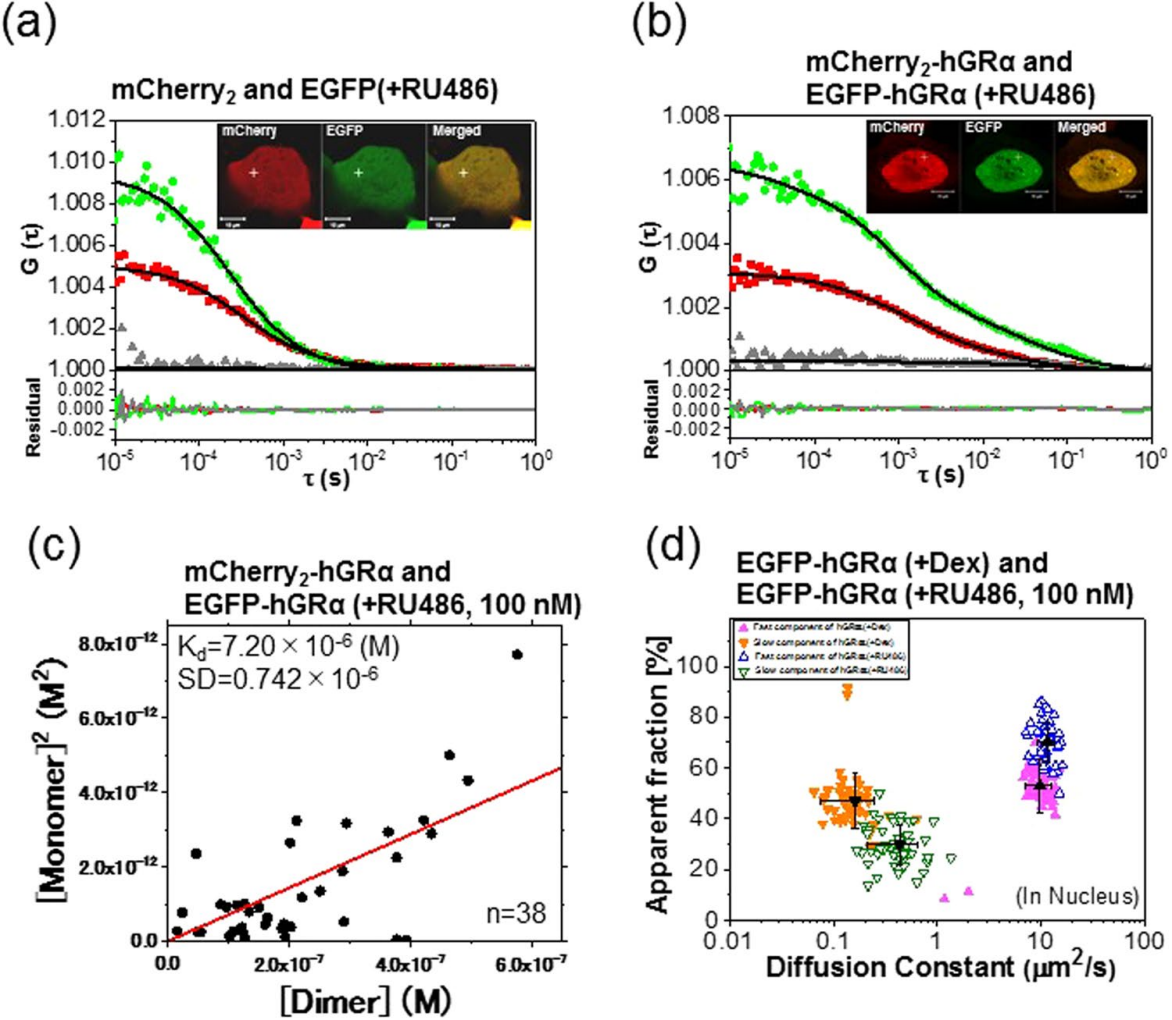
The effect of RU486 on GRα analyzed by FCCS. Typical auto- and cross-correlation curves obtained from U2OS cells coexpressing the pairs of chimeric fusion proteins before and after addition of the ligand. The filled green diamonds, red squares, and gray triangles denote autocorrelation of the green channel [*G*_*G*_(*τ*)], autocorrelation of the red channel [*G*_*R*_(*τ*)], and the cross-correlation curve [*G*_*C*_ *(τ*)], respectively, with their fits (solid black lines) and residuals. The insets show LSM images of U2OS cells coexpressing the pairs of chimeric fusion proteins. Measurement positions of FCCS are indicated by the white crosshairs. The scale bars are 10 μm. FCCS was performed in U2OS cells expressing (**a**) mCherry_2_ and EGFP as a negative control, after addition of RU486 and showing a flat cross-correlation amplitude; (**b**) U2OS cells coexpressing mCherry_2_-hGRα and EGFP-hGRα in the nucleus 20 min after addition of 100 nM RU486. (**c**) The K_d_ plot represents the square of the concentrations of the monomeric hGRα versus the concentration of the dimer of hGRα after addition of RU486. The solid line shows the linear fit. (**d**) The scatter plots represent the diffusion constants versus their fractions from fitting analysis of FCCS data with a two-component model. Black symbols indicate the average of the diffusion constants of the fast and slow components. The data are presented as mean ± SD. Fast and slow components are shown with different colors and symbols. EGFP-hGRα after addition of Dex (filled symbols) and RU486 (open symbols).

To create a model of inhibition of GRα homodimerization, cells coexpressing mCherry_2_-hGRα and EGFP-hGRα were incubated with RU486 (mifepristone), which is an inhibitor of transcription-regulatory activity^43, 44^. In the inset of Fig. 4(b), mCherry_2_-hGRα and EGFP-hGRα relocated to the nucleus after the addition of RU486, as with Dex. A negligible cross-correlation amplitude was occasionally observed (Fig. 4(b)), but the obtained K_d_ value was 7.20 μM (Fig. 4(c)), which was the same as that of the WT in the absence of Dex (7.40 μM) in the cytoplasm, suggesting that WT GRα had a tendency toward the monomer form during treatment with RU486. However, at the high concentration (1 μM) of RU486, the homodimerization of GRα in the presence of RU486 has been reported after a number and brightness analysis *in vivo* ^22^. K_d_ value was also determined at 1 μM RU486 treatment. Low cross-correlation amplitude was observed (Fig. S6A) and K_d_ value was 8.59 μM (Fig. S6B), which was similar to that in the presence of 100 nM RU486. The diffusion constant of EGFP-hGRα was determined by the autocorrelation function fitted to the two-component model, which also provided the dynamic properties of GRα before and after the addition of Dex. Figure S7 shows scatter plots of diffusion constants versus apparent fraction percentages of EGFP-hGRα in the absence and presence of Dex. The diffusion constant of the slow component decreased in the presence of Dex compared with its absence (Fig. S7). Overall, these results indicated a slowdown of GRα mobility in the presence of Dex for the complex formation with associated molecules and for interaction with DNA. Furthermore, the effect of the antagonist (RU486) on the diffusion of GRα was assessed in FCCS experiments (Fig. 4(d)). The diffusion constant of the fast component of WT GRα was not affected by the presence of RU486, in contrast to Dex. In addition, the diffusion constant of the slow component in the presence of RU486 became larger than that during Dex treatment, suggesting that the molecule became fast moving (Fig. 4(d), downward triangle). These results indicated dissociation of the initial complex and/or an unstable complex formation of GRα with the GRE in the presence of RU486, in agreement with our previous report^2^.

### FCCS analysis of the mutants of hGRα in the nucleus

**Figure 5:**
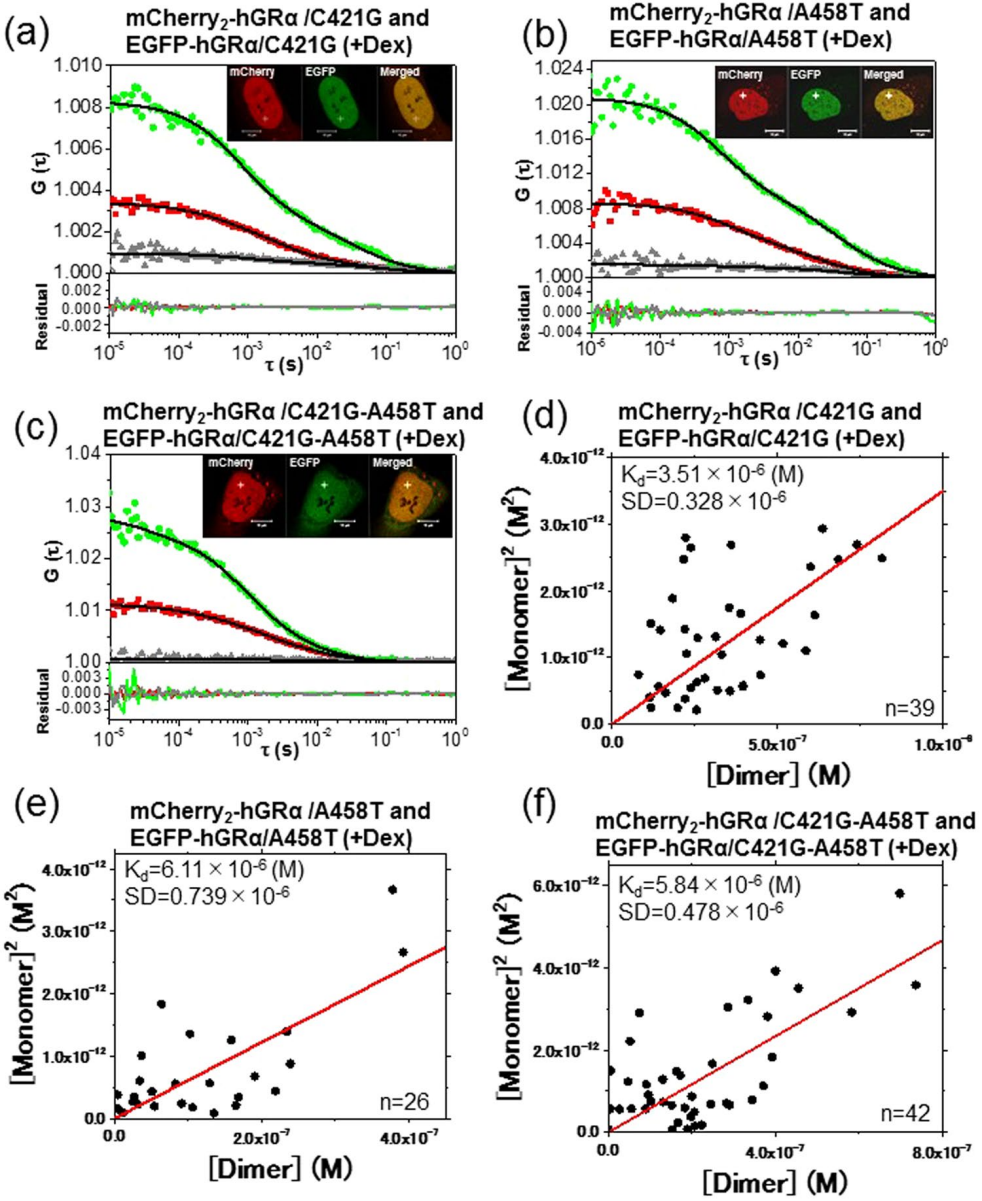
FCCS and K_d_ analysis of GRα mutants. Typical auto- and cross-correlation curves obtained from U2OS cells coexpressing the pairs of chimeric fusion proteins after addition of the ligand. The filled green diamonds, red squares, and gray triangles denote autocorrelation of the green channel [*G*_*G*_(*τ*)], autocorrelation of the red channel [*G*_*R*_(*τ*)], and cross-correlation curve [*G*_*C*_(*τ*)], respectively, with their fits (solid black lines) and residuals. The insets show LSM images of U2OS cells coexpressing the pairs of chimeric fusion proteins. FCCS analyses were performed in the nucleus, which is indicated by the white crosshairs. The scale bars are 10 μm. FCCS was conducted on U2OS cells coexpressing (**a**) mCherry_2_-hGRα**/**C421G and EGFP-hGRα**/**C421G, (**b**) mCherry_2_-hGRα**/**A458T and EGFP-hGRα**/**A458T, or (**c**) mCherry_2_-hGRα**/**C421G-A458T and EGFP-hGRα**/**C421G-A458T 20 min after addition of 100 nM Dex. (**d,e,f**) Results of K_d_ determination using scatter plots and linear regression. The plots represent the square of the concentration of the monomeric hGRα versus the concentrations of the dimer of hGRα. The solid lines show the linear fit. The slope indicates K_d_. (**d**) mCherry_2_-hGRα/C421G and EGFP-hGRα/C421G after addition of Dex. (**e**) mCherry_2_-hGRα/A458T and EGFP-hGRα/A458T after addition of Dex. (**f**) mCherry_2_-hGRα/C421G-A458T and EGFP-hGRα/C421G-A458T after addition of Dex.

To confirm the homodimerization of GRα in living cells, U2OS cells were transiently cotransfected with mCherry_2_-hGRα/C421G and EGFP-hGRα/C421G (Fig. S1C and D), mCherry_2_-hGRα/A458T and EGFP-hGRα/A458T (Fig. S1E and F), and with mCherry_2_-hGRα/C421G-A458T and EGFP-hGRα/C421G-A458T (Fig. S1G and H). According to the insets of Fig. 5(a), (b), and (c), C421G, A458T, and C421G-A458T were translocated to the nucleus after the addition of Dex as WT GRα. It should be noted that there was no difference in the static laser scanning microscopy (LSM) imaging method between the WT and mutants. The cross-correlation amplitude was observed in the C421G mutant (Fig. 5(a)) and A458T mutant (Fig. 5(b)) after the addition of Dex. In contrast, the C421G-A458T (Fig. 5(c)) mutant showed low cross-correlation amplitude in the nucleus after the addition of Dex. K_d_ values of mCherry_2_- and EGFP-fused C421G, A458T, and C421G-A458T were calculated from each slope: 3.51, 6.11, and 5.84 μM, respectively (Fig. 5(d)–(f)). These results suggested that the tendencies of the A458T and C421G-A458T mutants toward a monomer form were stronger than that of the WT (3.00 μM). In contrast, the tendency of C421G to homodimerization was similar to that of the WT. The scaffold effect of DNA for GR homodimerization was not significantly observed *in vivo*, which was observed *in vitro* experiments. Next, the diffusion properties of GRα mutants were analyzed in the nucleus of a live cell. The autocorrelation functions of EGFP-hGRα/C421G, EGFP-hGRα/A458T, and EGFP-hGRα/C421G-A458T mutants in the nucleus were analyzed by two-component fitting. Comparative analysis of the diffusion constants of WT GRα and its mutants are shown in scatter plots in Fig. S8. The fast component of each mutant was not affected by the addition of Dex. In contrast, the diffusion constants of the slow component increased after the addition of Dex, in comparison with the WT (Fig. S8).

### FCCS analysis of NLS region-mutated hGRα in the cytoplasm

**Figure 6:**
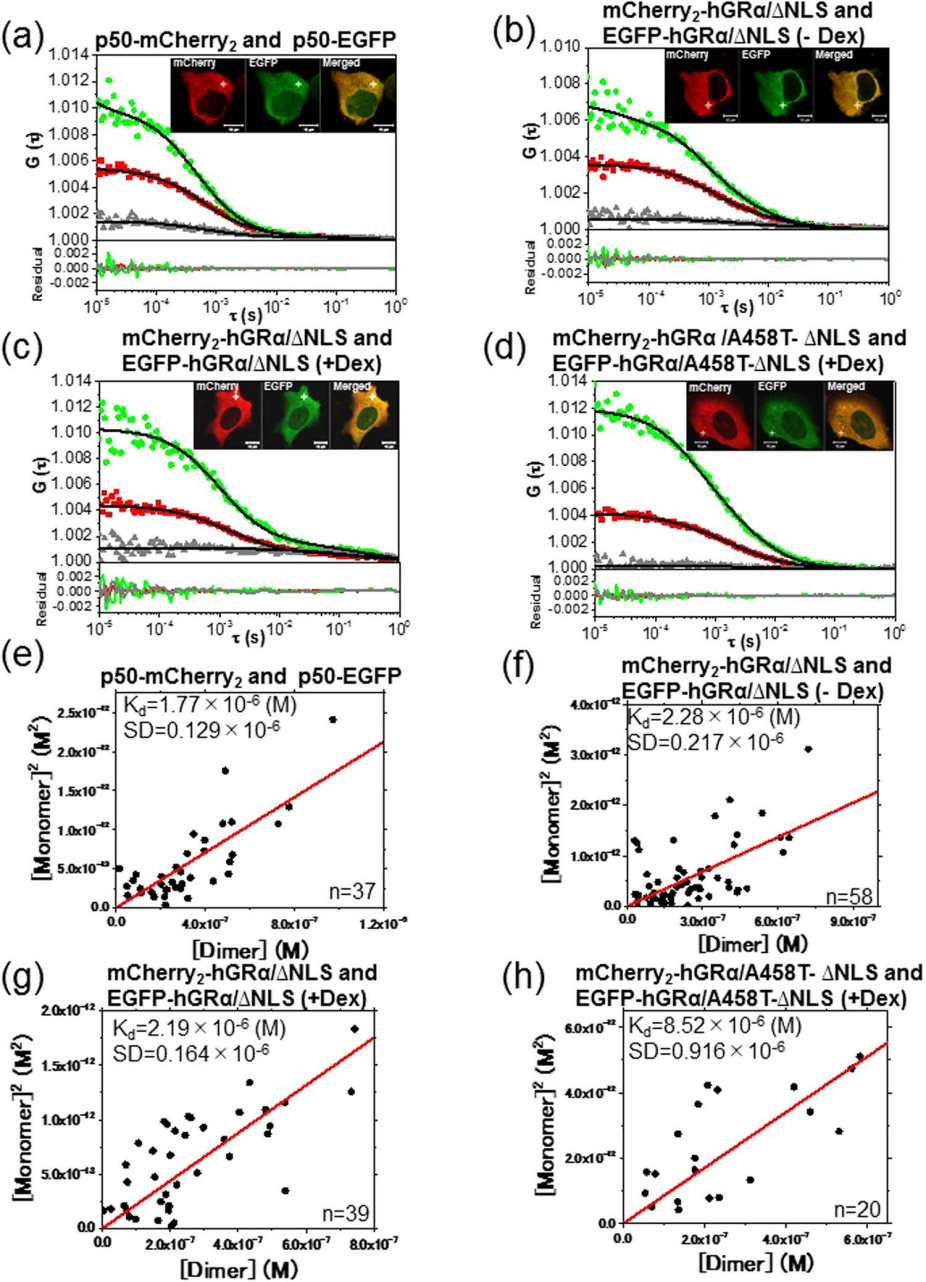
The effect of NLS mutation on formation of the GRα dimer. Typical auto- and cross-correlation curves obtained from U2OS cells coexpressing the pairs of chimeric fusion proteins before and after addition of the ligand. The filled green diamonds, red squares, and gray triangles denote autocorrelation of the green channel [*G*_*G*_(*τ*)], autocorrelation of the red channel [*G*_*R*_(*τ*)], and the cross-correlation curve [*G*_*C*_(*τ*)], respectively, with their fits (solid black lines) and residuals. The insets show LSM images of the U2OS cells coexpressing the pairs of chimeric fusion proteins. FCCS analyses were carried out in the cytoplasm, which is indicated by the white crosshairs. The scale bars are 10 μm. FCCS was performed using U2OS cells coexpressing (**a**) p50-mCherry_2_ and p50-EGFP as a positive control, (**b**) mCherry_2_-hGRα**/**∆NLS and EGFP-hGRα/∆NLS before addition of Dex, (**c**) mCherry_2_-hGRα**/**∆NLS and EGFP-hGRα/∆NLS 20 min after addition of 100 nM Dex, or (**d**) mCherry_2_-hGRα**/**A458T**-**∆NLS and EGFP-hGRα **/**A458T**-**∆NLS 20 min after addition of 100 nM Dex. (**e–h**) Results of K_d_ determination using scatter plots and linear regression. The plots represent the square of the concentration of the monomeric hGRα versus the concentrations of hGRα dimer. The solid line shows the linear fit. The slope indicates the K_d_. (**e**) p50**-**mCherry_2_ and p50-EGFP. (**f**) mCherry_2_-hGRα/∆NLS and EGFP-hGRα/∆NLS before addition of Dex. (**g**) mCherry_2_-hGRα/∆NLS and EGFP-hGRα/∆NLS after addition of Dex. (**h**) mCherry_2_-hGRα/A458T-∆NLS and EGFP-hGRα/A458T-∆NLS after addition of Dex.

**Figure 7:**
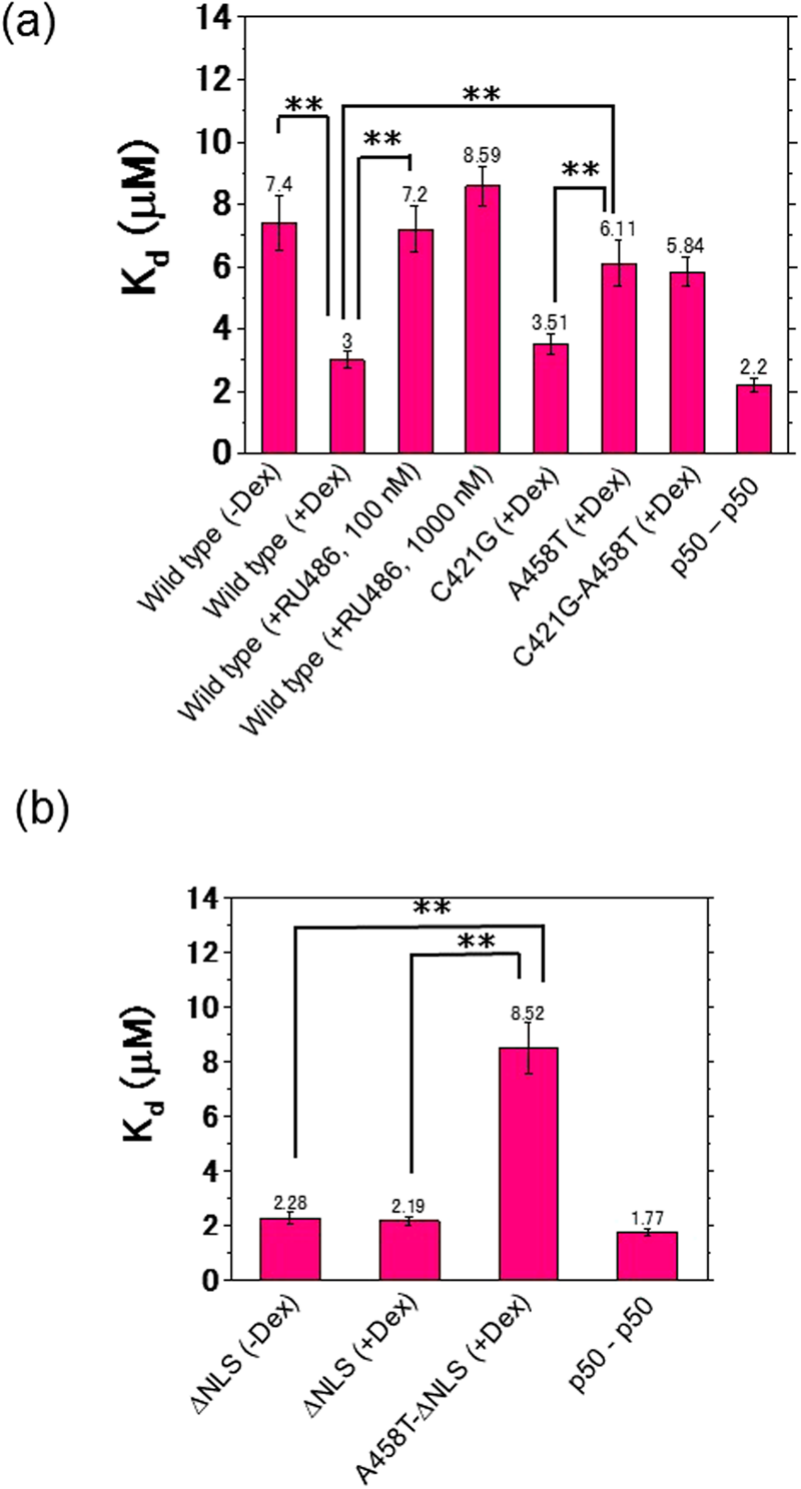
A summary of the dissociation constants (K_d_) of the WT and mutants before and after addition of ligands. The bars indicate the dissociation constants, K_d_. (**a**) Before addition of Dex, the dissociation constant (K_d_) of the WT was 7.40 μM in the cytoplasm, and after addition of Dex the K_d_ values of the WT, C421G, A458T, and C421G-A458T were 3.00, 3.51, 6.11, and 5.84 μM, respectively, in the nucleus. K_d_ of the control p50-p50 in the nucleus was 2.20 μM. (**b**) Before addition of Dex, K_d_ of ∆NLS was 2.28 μM and after addition of Dex, K_d_ values of the ∆NLS and A458T-∆NLS mutants in the cytoplasm were 2.19 and 8.52 μM, respectively. K_d_ of the p50-p50 dimer in the cytoplasm was 1.77 μM. The data are presented as mean ± SD. Statistical analysis was based on ANOVA (*p < 0.05, **p < 0.01).

To test whether the GRα homodimerizes in the cytoplasm, we constructed mCherry_2_- and EGFP-fused nuclear localization signal 1-mutated (∆NLS) hGRα that did not relocate to the nucleus, and A458T-∆NLS mutants that neither formed homodimers nor relocated to the nucleus. U2OS cells were transiently cotransfected with plasmids encoding mCherry_2_-hGRα/∆NLS and EGFP-hGRα/∆NLS (Fig. S1I and J) and mCherry_2_-hGRα/A458T-∆NLS and EGFP-hGRα/A458T-∆NLS (Fig. S1K and L). As a positive control, FCCS was conducted on p50-mCherry_2_ and p50-EGFP (Fig. S1O and P), which were coexpressed in the cytoplasm (Fig. 6(a), inset). K_d_ values were found to be 1.77 μM for p50-mCherry_2_ and p50-EGFP in the cytoplasm (Fig. 6(e)). According to the insets of Fig. 6(c) and (d), the mCherry_2_- and EGFP-fused ∆NLS mutant and the A458T-∆NLS mutant were localized to the cytoplasm in the presence of Dex. FCCS was performed in the cytoplasm in the absence (Fig. 6(b)) and presence of Dex (Fig. 6(c)). Unexpectedly, a cross-correlation amplitude was observed in both Fig. 6(b) and Fig. 6(c). K_d_ values of mCherry_2_-hGRα/∆NLS and EGFP-hGRα/∆NLS in the absence and presence of Dex were found to be 2.28 μM (Fig. 6(f)) and 2.19 μM (Fig. 6(g)), respectively. Our results suggested that in the condition without Dex stimulation, GRα/∆NLS had a lesser tendency toward monomerization but also formed a homodimer in the cytoplasm, whereas the proportion of GRα homodimers tended to increase after the addition of Dex. To confirm the GRα homodimerization in the cytoplasm, FCCS of the homodimerization-deficient mutant, mCherry_2_-hGRα/A458T-∆NLS, and EGFP-hGRα/A458T-∆NLS was performed in the presence of Dex (Fig. 6(d)). Very low cross-correlation amplitude was only observed. K_d_ was found to be 8.52 μM (Fig. 6(h)), which was higher than that of EGFP-hGRα/ΔNLS and mCherry_2_-hGRα/ΔNLS in the absence and presence of Dex. Figure 7(a) and (b) show summaries of the obtained K_d_ values in living cells. This evidence pointed to the presence of the homodimer of GRα in the cytoplasm. The diffusion properties of these mutants were analyzed in the cytoplasm of living cells. Comparative analysis of the diffusion constants of the WT and mutants is shown in scatter plots in Fig. S9. In the cytoplasm, the fast and slow components of each mutant were not affected by the addition of Dex. This observation was suggestive of formation of the complex between the NLS-mutated GRα and other cytoplasmic proteins, same as WT GRα. Taken together, these results supported the hypothesis that GRα can homodimerize in the cytoplasm in the presence of Dex.

## Discussion

**Figure 8:**
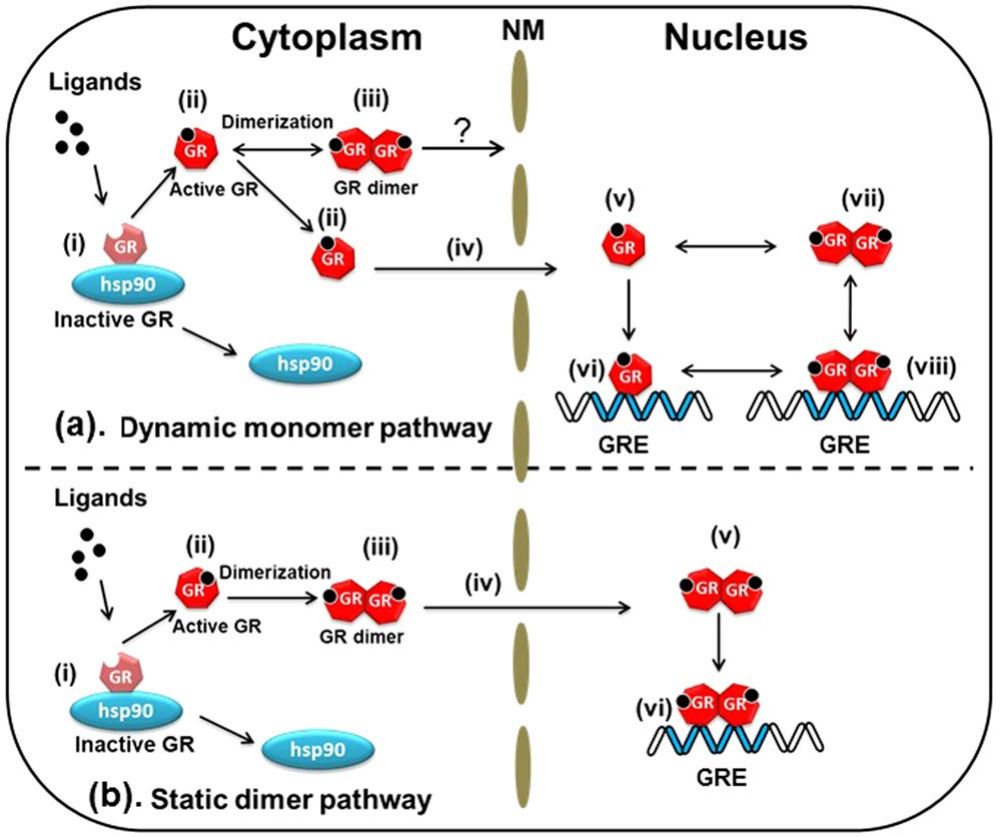
The proposed model for the pathways of glucocorticoid receptors. (**a**) The dynamic monomer pathway: (i) hGRα is localized to the cytoplasm as a complex or in free form in the uninduced state. (ii) hGRα is activated after ligand binding. Activated hGRα in the cytoplasm is in equilibrium between a monomer and dimer (iii) but transport of dimeric hGRα is unclear. (iv) Activated monomer hGRα relocates into the nucleus and is in both the free state (v) and monomer form, which can bind to a GRE as an unstable complex (vi). (vii) hGRα further dimerizes in the nucleus. (viii) The preformed dimer of hGRα associates with the GRE and other transcription factors. The dimer and monomer are not only distributed in the cytoplasm but also in the nucleus even after ligand binding; however, transport of hGRα is carried out in the monomeric form of hGRα. The concentration of hGRα in the nucleus can be controlled by changing the K_d_ of hGRα and GRE in the nucleus. (**b**) The static dimer pathway: (i) hGRα is localized to the cytoplasm as a complex or in free form in the uninduced state. (ii) hGRα is activated after ligand binding. Activated hGRα exists in the cytoplasm as a dimer (iii). (iv) In the dimer form, hGRα is translocated. The preformed dimer (v) of hGRα associates with a GRE and transcription factors (vi). The dimer of hGRα is distributed in both the cytoplasm and nucleus, but the monomer is found only in the cytoplasm. hGRα is transported in the dimer form. The concentration of hGRα in the nucleus can be controlled by the activity and functions of the NPC. hGRα: human glucocorticoid receptor α, hsp90: heat shock protein 90, GREs: glucocorticoid response elements, NM: nuclear membrane, NPC: nuclear pore complex

As mentioned in the introduction, there are a number of controversial issues regarding homodimerization of GRα. Generally, steroid receptors regulate transcription via two main pathways. In the first one, two molecules of steroid receptors bind to DNA in a cooperative manner; thus, binding of the first molecule accelerates the binding of the second molecule sequentially, forming a homodimer via a dimerization interface (*monomer pathway*). In the other pathway, preformed homodimers of steroid receptors bind to DNA (*dimer pathway*)^22, 45–48^. In the case of GRα, it is still a subject of debate when and where the homodimerization takes place and whether it proceeds through the monomer pathway^10, 15, 16, 49^ or the dimer pathway^11, 17–21, 50^. Our observations and other studies indicate that the transition time from the cytoplasm to the nucleus ranges from 10 to 60 min after the addition of Dex^2, 51, 52^. We can hypothesize a dynamic monomer pathway where GRα is in equilibrium between monomeric and homodimeric forms in the cytoplasm as well as in the nucleus during this rather long transition time, and where GRα relocates to the nucleus as a monomer and forms the GRα homodimer before DNA binding in the nucleus (Fig. 8).

K_d_ of homodimerization of the WT and mutants of GRα *in vitro* was confirmed using the FCCS-microwell system. The *in vitro* K_d_ of WT GRα was determined to be 416 and 139 nM in the absence and presence of Dex, respectively (Fig. 1(e) and (f)). This result is in good agreement with K_d_ obtained in the brightness analysis using the FCS-microwell system^14^. Moreover, K_d_ for homodimerization of the C421G mutant and A458T mutant *in vitro* was 244 and 379 nM, respectively, in the presence of Dex (Fig. 2(e) and (f)). The tendencies of K_d_ values of the WT relative to the mutants *in vitro* were similar to those in living cells (Figs 2(g) and 7(a)). On the other hand, the absolute values of K_d_ *in vitro* were lower than those in living cells. This finding suggests that there are some mechanisms that keep the monomer form of hGRα in living cells.

Next, K_d_ values of hGRα homodimerization were determined by FCCS in living cells. K_d_ of WT GRα was 7.40 μM in the cytoplasm in the absence of Dex (Fig. 3(f)) and 3.00 μM in the nucleus in the presence of Dex (Fig. 3(g)), indicating that GRα has a tendency toward homodimerization in the nucleus (Fig. 8(a), vii, viii). By contrast, K_d_ of the GRα homodimerization was in the nanomolar range *in vitro* (Fig. 2(g)). Our findings indicate that GRα forms a homodimer in the nucleus in the presence of Dex; however, judging by the rather high value of K_d_ (3.00 μM), GRα in equilibrium is distributed between the monomeric form and homodimeric form (Fig. 8(a), v↔vii). The presence of both monomeric and homodimeric forms at the rather high value of K_d_ than expression level of hGRα in the living cells (300 nM to 2300 nM for WT hGRα) may enable formation of complex with other nuclear receptors such as mineralocorticoid receptor. K_d_ of the A458T mutant (homodimerization-deficient mutant^39^) was 6.11 μM in the presence of Dex (Fig. 5(e)), which is higher than that of WT GRα in the presence of Dex (3.00 μM) and lower than that of WT GRα in the absence of Dex (7.40 μM). The tendency of the A458T mutant toward the monomer was stronger than that of WT GRα in the presence of Dex (Fig. 7(a)). These results indicate that the A458T mutation in GRα impairs the homodimerization in living cells, but some part of the A458T mutant forms a homodimer. This finding is consistent with the literature data, which suggest that the A458T mutant can form a homodimer^53^.

In the present study, effects of RU486 on the process of hGRα homodimerization were also examined. It has been demonstrated that RU486 works as an antagonist of transcription-regulatory activity^43, 44^, but some studies revealed its partial agonist behavior for transcription of GRα^22, 54–56^. K_d_ of WT GRα was 7.20 μM and 8.59 μM in the presence of 100 nM and 1000 nM RU486, respectively (Figs 4(c) and S6); these values are the similar to that of the WT in the cytoplasm in the absence of Dex (7.40 μM). This observation suggests that a lack of homodimer of GRα inhibits the transcriptional-regulatory activity. In contrast, homodimerization of GRα in the presence of RU486 has been reported after a number and brightness analysis *in vivo* ^22^. This discrepancy is unclear, but may be due to the different cell line with the expression of GRα in the living cells.

Our results also answered the question whether binding of GRα to DNA is necessary for homodimerization. The C421G mutant, which cannot associate with a GRE, does not have a transcription-regulatory activity^38^. K_d_ of the C421G-A458T double mutant of GRα was 5.84 μM in the presence of Dex (Fig. 5(f)); this value was higher than that of the C421G mutant but was the same as that of A458T mutant in the presence of Dex. Some studies suggested that GRα homodimerizes only after DNA binding^10, 16, 49^. In contrast, earlier studies had revealed GRα homodimerization in solution, independently of DNA binding^14, 23^. Our results support GRα homodimerization before DNA binding (Fig. 8(a), vii) according to FCCS measurement of the DNA-binding-deficient mutant (C421G) and a double mutant with DNA-binding and dimerization deficiencies (C421G-A458T; Fig. 7(a)). To reconcile these discrepancies, however, a more dynamic view of hGRα is needed.

K_d_ of the A458T mutant and C421G-A458T mutant was higher than that of C421G and lower than those of the WT (without Dex) (Fig. 7(a)). Thus, these results suggest that C421G-A458T and A458T has a tendency to be in the monomeric form but also in the homodimeric form. Moreover, different diffusion properties were observed between A458T and C421G-A458T (Fig. S8E). The diffusion constant of the slow component of the C421G-A458T mutant was greater (faster) than that of A458T in the presence of Dex (Fig. S8E). This finding suggests that C421G-A458T cannot bind to a GRE but A458T can do so as a monomer (Fig. 8(a), v→vi). This result seems to support the finding that monomers of WT GRα and of the A458T mutant have a weak transcription-regulatory activity in a reporter assay involving a palindromic GRE sequence^57^. These data suggest that an initial and/or unstable complex of the A458T GRα mutant with the GRE forms in the presence of Dex (Fig. 8(a), v→vi), in line with our previous reports^2, 51^.

To test whether GRα homodimerizes in the cytoplasm, the K_d_ values of ∆NLS and A458T-∆NLS mutants of GRα in the presence of Dex were determined by FCCS. These mutants were expected to be incapable of translocation to the nucleus or formation of homodimers in the presence of Dex. We found the K_d_ values of ∆NLS to be 2.28 μM in the absence of Dex (Fig. 6(f)) and 2.19 μM in its presence (Fig. 6(g)). These data are suggestive of the presence of a preformed homodimer in the unliganded state in the cytoplasm because K_d_ was lower than that of the WT GRα in the absence of Dex. It is possible that the mutation of NLS elicits a conformational change and/or a big change in electrostatic properties of the GRα moieties that facilitates formation of homodimers. In contrast, K_d_ of the A458T-∆NLS mutant was estimated to be 8.52 μM (Fig. 6(h)). These findings reinforce the idea that cytoplasmic homodimerization of GRα takes place at the initial stage of stimulation (Fig. 8(a), ii→iii) in agreement with other studies^17, 23^. As expected, the tendency of the A458T-ΔNLS mutant toward the monomeric state is stronger than that of the ∆NLS mutant after the addition of Dex. However, the results do not support the notion that GRα relocates into the nucleus in homodimeric form because K_d_ is still in the micromolar range, i.e., much higher than the *in vitro* K_d_ values. It can thus be reasonably assumed that the K_d_ value should be in the nanomolar concentration range if all of GRα form a homodimer after ligand binding. Therefore, our results do not support the translocation of GRα from the cytoplasm to the nucleus as a homodimer (Fig. 8(a), iii and iv) although a recent study showed the translocation of GRα in the homodimer form^17^. Further experiments, such as single-molecule tracking or multipoint FCCS need to be carried out to uncover the details of quaternary structure during the transport through the nuclear pore; we would like to do these experiments in a future study.

There may be a static dimer pathway, where GRα is transported from the cytoplasm to the nucleus in the homodimeric form. Thus, the concentration of GRα in the nucleus can be controlled by activity of the nuclear pore complex (Fig. 8(b), NPC). In contrast, our results support the existence of a dynamic monomer pathway, in which the concentration of GRα in the nucleus can be controlled by changes in the binding affinity between GRα and a GRE (Fig. 8(a)). Our findings appear to be substantiated by a report on a mineralocorticoid receptor (MR) indicating that only homodimers that form in the nucleus (after activation by ligand binding) can be transcriptionally active, whereas homodimers in the cytoplasm do not have the ability to relocate to the nucleus or regulate gene expression^45^.

In conclusion, our quantitative data show homodimerization of hGRα in the nucleus and cytoplasm of living cells. To our knowledge, this is the first report of quantitative differences between homodimerization of WT GRα and homodimerization of its mutants on the basis of dissociation constants. The evidence obtained in this study suggests that DNA binding is not necessary for GRα homodimerization in the nucleus *in vivo*. Our findings should advance the understanding of the homodimerization, DNA binding, and dynamics of GRα in living cells.

## Materials and Methods

### Chemicals and antibodies

Dexamethasone (Dex) and RU486 were purchased from Sigma-Aldrich. McCoy’s 5A modified medium and charcoal-stripped fetal bovine serum were purchased from GIBCO (Invitrogen). A mouse monoclonal anti-GR antibody (ab9568) was acquired from Abcam; a monoclonal anti-GFP (mouse IgG1-K) antibody (GF200) from Nacalai Tesque, Inc.; anti-actin clone C4 (mouse monoclonal) antibody from Millipore; an anti-NF-κBp50 (D-6) sc-166588 mouse monoclonal IgG1 antibody from Santa Cruz Biotechnology, and the alkaline phosphatase-conjugated anti-mouse antibody was purchased from Biosource^TM^.

### Plasmids

All schematic representations of the plasmids are shown in Fig. S1. The plasmids encoding human glucocorticoid receptor α (hGRα) fused with EGFP, its mutants A458T and C421G were described elsewhere^2^. The pEGFP-hGRα/C421G-A458T was constructed by insertion of the fragment amplified from DNA with the A458T mutation^2^ into pEGFP-hGRα/C421G as a vector with restriction enzymes Esp3I and ClaI. For ∆NLS mutation (K494A, K495A, and K496A), a first-step PCR was performed using the following primers:

Forward-1: 5′-gggtccccaggtaaagagacgaa-3′ and

Reverse-1: 5′-ccttttatggcggcggctgtttttcgagcttc-3′ and

Forward-2: 5′-cgaaaaacagccgccgccataaaaggaattcag-3′ and

Reverse-2: 5′-agaaacatccaggagtactgcagtaggg-3′

with pEGFP-hGRα and pEGFP-hGRα/A458T as a template. Then the first-step PCR products were mixed as a template, and second-step PCR was performed with the above forward-1 and reverse-2 primers. The second-step PCR product was digested with Esp3I and PstI, and ligated into pEGFP-hGRα as a vector that was digested with the same restriction enzymes.

To construct the mCherry tandem dimer (mCherry_2_)-fused hGRα, the fragment encoding EGFP was swapped for the fragment encoding mCherry_2_ by digestion with AgeI and Bsp1407I and ligation with the “Mighty mix” DNA ligation kit (Takara, Japan). To construct the mCherry_2_-fused hGRα mutants (C421G, A458T, C421G-A458T, ∆NLS, and A458T-∆NLS), the hGRα in pmCherry_2_-hGRα was swapped for the hGRα containing each mutation with ScaI and Bsp1407I and ligation with the “Mighty mix” DNA ligation kit.

As a positive control, we used the well-known p50 protein, a subunit of NF-κB. We constructed a plasmid encoding the IPT (immunoglobulin-like plexin transcription factor) domain of p50 fused with the N terminus of mCherry_2_ or EGFP (Fig. S10). For localization of p50 to the nucleus, p50-mCherry_2_/NLS and p50-EGFP/NLS were constructed. The SV40 large T antigen NLS (Pro-Lys-Lys-Lys-Arg-Lys-Gly) fused with the C-terminal mCherry_2_ or EGFP and the p50 fragment flanked by NheI and AgeI sites were inserted into the N-terminal pmCherry_2_/NLS or pEGFP/NLS, then digested at the same restriction sites. As a negative control, we used a plasmid encoding mCherry_2_ and EGFP.

### Cell culture and transient transfection

U2OS cells were maintained in a humidified atmosphere containing 5% CO_2_ at 37 °C in McCoy’s 5A modified medium supplemented with 10% charcoal-stripped fetal bovine serum, 100 U/mL penicillin G and 100 μg/mL streptomycin. For FCCS, U2OS cells were plated on a Lab-TeK^®^ 8-well chamber cover glass (Nunc^TM^) and cotransfected with different fusion constructs where the ratio of the amounts of the two plasmids was kept at 2:1 (200 ng**/**well pmCherry_2_ **-**hGRα and 100 ng**/**well pEGFP-hGRα) using Optifect^TM^ (Invitrogen). After 16 hrs of transfection, Dex or RU486 was added to each well at a final concentration of 100 nM with further incubation for 20 min at 37 °C.

### Western blotting

One day before transient transfection, U2OS cells (10^5^/well) were seeded on a 6-well Nunclon^TM^∆ chamber (Nalge Nunc International). Cells were transiently transfected with the transfection reagent (mock) alone or with 1 μg**/**well pEGFP-hGRα, its mutants, or p50-EGFP using Lipofectamine^TM^ 2000. After 4 hrs of transfection, the medium was replaced with a fresh one. Twenty-four hours after transfection, cells were washed with ice-cold PBS, trypsinized, collected in PBS containing trypsin inhibitor 4-[2-aminoethyl]benzenesulfonyl fluoride hydrochloride (Sigma-Aldrich), and centrifuged. The cell pellets were lysed in CelLytic^TM^ M lysis buffer (Sigma-Aldrich) supplemented with 1% protease inhibitor cocktail (Sigma-Aldrich). The homogenates were centrifuged (15000 rpm, 4 °C) for 10 min, and the cell lysates were collected. The lysates were solubilized with 2 × Laemmli sample buffer (Nacalai Tesque), heat denatured at 65 **°**C for 15 min, electrophoresed in a precast 7.5% polyacrylamide gel (ePAGEL, ATTO), and then transferred onto a PVDF membrane (Bio-Rad Laboratories, Hercules, CA). The membranes were blocked overnight in 5% skim milk and washed three times in PBST buffer (137 mM NaCl, 2.7 mM KCl, 10 mM Na_2_HPO_4_, 2 mM KH_2_PO_4_, pH 7.4, 0.05% Tween 20) at room temperature and incubated with the primary antibodies: anti-GR, anti-GFP, anti-actin, and anti-NF-κB p50 (1:1000 dilution in “Can Get Signal” Solution I; TOYOBO) for 1 hr at room temperature. After three washes in PBST, the membranes were incubated with an alkaline phosphatase-conjugated anti-mouse IgG antibody (secondary antibody, 1:1000 dilution in “Can Get Signal” solution II; TOYOBO) for 1 hr at room temperature. Then, the membranes were washed three times with PBST and reacted with an alkaline phosphatase substrate (SIGMA FAST^TM^ BCIP^®^ **/**NBT) solution.

### Microscopy and FCCS

Live-cell fluorescence imaging and FCCS measurements were performed by a LSM 510-ConfoCor3 (Carl Zeiss), which contained Ar^+^ laser and He-Ne laser, a water immersion objective (C-Apochromat, 40x, 1.2NA; Carl Zeiss), and two avalanche photodiodes. This setup was used both for FCCS and LSM imaging. The pinhole diameter was adjusted to 70 μm. EGFP and mCherry were excited by the 488-nm (15 μW) and 594-nm (8 μW) lasers, respectively. The emission signals were split by a dichroic mirror (600-nm beam splitter) and detected at 505–540 nm for EGFP and at 615–680 nm for mCherry. FCCS was performed 10 times with duration of 5 s before and 20 min after addition of the indicated ligands.

### Data analysis

FCCS data were analyzed by AIM software (Carl Zeiss). The autocorrelation functions from the green and red channels, *G*_*G*_(*τ*) and *G*_*R*_(*τ*), and the cross-correlation function, *G*_*C*_(*τ*), were computed as follows:

1 $${G}_{G}(\tau )=1+\frac{\langle \delta {I}_{G}(t)\cdot \delta {I}_{G}(t+\tau )\rangle }{\langle {I}_{G}(t)\rangle \cdot \langle {I}_{G}(t)\rangle }$$

2 $${G}_{R}(\tau )=1+\frac{\langle \delta {I}_{R}(t)\cdot \delta {I}_{R}(t+\tau )\rangle }{\langle {I}_{R}(t)\rangle \cdot \langle {I}_{R}(t)\rangle }$$

3 $${G}_{C}(\tau )=1+\frac{\langle \delta {I}_{G}(t)\cdot \delta {I}_{R}(t+\tau )\rangle }{\langle {I}_{G}(t)\rangle \cdot \langle {I}_{R}(t)\rangle }$$

where *τ* denotes the delay time; *I*_*G*_ and *I*_*R*_ are the fluorescent intensity of the green and red channels, respectively; and *G*_*G*_(*τ*), *G*_*R*_(*τ*), and *G*_*C*_(*τ*) denote the autocorrelation functions of green, red channels and cross-correlation function, respectively. The acquired auto- and cross-correlation functions were fitted to a two-component model^58^:

4 $$\begin{array}{rcl}G(\tau ) & = & 1+\frac{1-{F}_{triplet}+{F}_{triplet}\exp (-\tau /{\tau }_{trplet})}{N(1-{F}_{triplet})}\\  &  & \times ((\frac{{F}_{fast}}{1+\tau /{\tau }_{fast}})\sqrt{\frac{1}{1+\tau /{s}^{2}{\tau }_{fast}}}+(\frac{{F}_{slow}}{1+\tau /{\tau }_{slow}})\sqrt{\frac{1}{1+\tau /{s}^{2}{\tau }_{slow}}})\end{array}$$

where *F*_*triplet*_ is the average fraction of triplet state molecules, *τ*_*triplet*_ is the triplet relaxation time, *F*_*fast*_ and *F*_*slow*_ are the fractions of the fast and slow components, respectively, and *τ*_*fast*_ and *τ*_*slow*_ are the diffusion times of the fast and slow components, respectively. For cross-correlation fitting, the triplet was not used. *N* is the average number of fluorescent particles in the excitation-detection volume defined by ω_1_ and ω_2_ which are a radius of the short and long axis of the confocal volume, and *s* is the structural parameter representing the ratio *s* = ω_2_ **/**ω_1_. The values of ω_1,*i*_ (*i* = *G* or *R*) are calculated from the diffusion coefficients of rhodamine 6 G and Alexa 594 used as standard dyes, respectively.

5 $${\omega }_{1,i}=\sqrt{4D\cdot {\tau }_{Di}}$$

The volume elements *V* are calculated according to

6 $${V}_{i}={\pi }^{3/2}\cdot {\omega }_{1,i}^{2}\cdot {\omega }_{2,i}$$

7 $${V}_{C}={(\frac{\pi }{2})}^{3/2}({\omega }_{1,G}^{2}+{\omega }_{1,R}^{2}){({\omega }_{2,G}^{2}+{\omega }_{2,R}^{2})}^{1/2}$$

The apparent total numbers of autocorrelation particles *N*_*G*_ and *N*_*R*_ and of complex cross-correlated particles *N*_*C*_ are given in the case which brightness of fluorescent protein is homogenous by

8 $${N}_{G}=\frac{1}{{G}_{G}(0)-1}$$

9 $${N}_{R}=\frac{1}{{G}_{R}(0)-1}$$

10 $${N}_{C}=\frac{{G}_{C}(0)-1}{({G}_{R}(0)-1)\cdot ({G}_{G}(0)-1)}$$

When *N*_*G*_ and *N*_*R*_ are constant, *G*_*C*_ (*0*) is directly proportional to *N*_*C*_. The backgrounds of the resulting number of particles were corrected by subtracting autofluorescence intensity of mock-transfected U2OS cells, as follows^59^:

11 $${N}_{i,corrected}={N}_{i,measured}\cdot {[1-\frac{{I}_{i,background}}{{I}_{i,measured}}]}^{2}$$

Then,

12 $${N}_{C,corrected}=({G}_{C}(0)-1)\cdot {N}_{G,corrected}\cdot {N}_{R,corrected}$$

Diffusion constants of the samples were calculated from the ratio of the diffusion constant of rhodamine 6 G (*D*_*Rh6G*_; 414 μm^2^/s) and diffusion time τ_R6G_ and τ_Sample_^60^.

The apparent concentration of each fluorescent protein was calculated with A (Avogadro’s number) as shown below:

13 $$[{C}_{i,corrected}]=\frac{{N}_{i,corrected}}{{V}_{i}\cdot A}$$

14 $$[{C}_{C,corrected}]=\frac{{N}_{C,corrected}}{{V}_{C}\cdot A}$$

In actual measurement of EGFP-hGRα and mCh_2_-hGRα, monomer, homo-color dimer and hetero-color dimer were present in the living cells and lysate. The particle brightness of homo-color dimer was twice higher than that of monomer and hetero-color dimer. The square of average brightness of monomer, hetero-color dimer and homo-color dimer was contributed to the amplitude of autocorrelation functions. Therefore, their concentrations were calculated using relative values of particle brightness of EGFP-hGRα and mCh_2_-hGRα against EGFP and mCherry_2_ co-expression sample (See supplemental information).

### Determination of K_d_

The dissociation constant K_d_ was determined using the following equations:

15 $${K}_{d}=\frac{{[{\rm{M}}]}^{2}}{[{\rm{D}}]}=\frac{{([{\rm{G}}]+[{\rm{R}}])}^{2}}{[{\rm{GG}}]+[{\rm{RR}}]+[{\rm{RG}}]}$$

[M] and [D] show the concentration of monomeric hGRα and dimeric hGRα, respectively. In the cells, EGFP-hGRα and mCh_2_-hGRα were expressed. Therefore, [M] and [D] was transformed to [G] + [R] and [GG] + [RR] + [RG], respectively. G and R denotes the EGFP-hGRα and mCh_2_-hGRα. The concentration of hetero-color dimer, [RG] was calculated from the cross-correlation amplitude. Monomers and hetero-color dimers, [G] + [RG] and [R] + [RG] and homo-color dimers, [GG] and [RR] were calculated using relative values of particle brightness of EGFP-hGRα and mCh_2_-hGRα against EGFP and mCherry_2_ co-expression sample (See supplemental information), because particle brightness of homo-color dimer is twice higher than monomeric GR and hetero-color dimer. Taken together with concentration of hetero-color dimer [RG] calculated from the cross-correlation amplitude, concentrations of monomers ([G] and [R]), homo-color dimers ([GG] and [RR]) and hetero-color dimer [RG] were separately determined. According to the simulation result, the measured K_d_ values were completely matched to the given K_d_ values by the K_d_ calculation method with the concentration of homo-color dimer, but were not matched without its consideration (Fig. S14). The relative cross amplitudes (RCA) *in vitro* and *in vivo* were significantly higher than that of coexpression of EGFP and mCherry_2_ as a negative control. Moreover, fold change of RCA values against negative control was over 5.6 for *in vitro* experiments and 17 for *in vivo* experiments (Figs S4 and S5), suggesting that the background cross-correlation amplitude, such as cross-talk signal is not dramatically affected to cross-correlation amplitude of interactions of EGFP-hGRα and mCh_2_-hGRα. Some data points in which concentrations of monomer ([G] or [R]) or homo-color dimer ([GG] or [RR]) show minus values due to experimental errors were excluded from K_d_ determination (Figs S11 and S12). Then a scatter plot of the products of concentrations of monomeric GR ([M] = [G] + [R]) versus the concentration of the dimeric GR ([D] = [GG] + [RR] + [RG]) was generated with a line of best fit, and the K_d_ was calculated from the slope of the regression line^30, 32^. All data points were strongly correlated between the square of the concentration of monomeric GR and the concentration of dimeric GR (Fig. S13).

### Determination of *in vitro* K_d_ by FCCS-microwell system

U2OS cells were cotransfected with 2 μg pmCherry_2_-hGRα and 1 μg pEGFP-hGRα using ViaFect^TM^ (Promega). The culture method for microwell and extraction method of hGRα from the nucleus were described previously^14^. The optical setup for FCCS was the same as for *in vivo* FCCS. The power of 488-nm and 594-nm lasers was 15 and 12 μW, respectively. The data analysis and computation of K_d_ values of hGRα were the same as for *in vivo* FCCS.

## Electronic supplementary material

Supplemental information
