# Supplementary material for: A Quantitative Study of Internal and External Interactions of Homodimeric Glucocorticoid Receptor Using Fluorescence Cross-Correlation Spectroscopy in a Live Cell
Source: Sci Rep. 2017 Jun 28;7:4336. doi: 10.1038/s41598-017-04499-7 (PMC5489515; doi:10.1038/s41598-017-04499-7)
Supplement: Supplementary file 1 — Supplemental information [file 41598_2017_4499_MOESM1_ESM.pdf]

A Quantitative Study of Internal and External Interactions of Homodimeric Glucocorticoid Receptor Using Fluorescence Cross-Correlation Spectroscopy in a Live Cell

Manisha Tiwari[†], Sho Oasa[†], Johtaro Yamamoto, Shintaro Mikuni, and Masataka Kinjo*

Laboratory of Molecular Cell Dynamics,

Faculty of Advanced Life Science,

Hokkaido University,

Sapporo 001-0021 Japan.

*To whom correspondence should be addressed: Laboratory of Molecular Cell Dynamics, Faculty of Advanced Life Science, Hokkaido University, N21W11, Kita-ku, Sapporo 001-0021, Japan.

† These authors made an equal contribution.

Supplemental method for K_d determination.

1. Number of GR monomer and dimer

ACF of FCS for several species with different brightness can be expressed as

$$G(\tau) = \frac{\sum N_i B_i^2 D_i(\tau)}{\left(\sum N_i B_i\right)^2}, \tag{1}$$

where N_i, B_i, and D_i indicate the number of molecules, molecular brightness, and decay curve due to translational diffusion of *i*-th species [Lakowicz, p895, eq(24.19)]. In our experiment, we assumed the decay curve of fluorescently labeled GR monomer and GR dimer could not be separated because the diffusion time of GR monomer and dimer take close value since the molecular weight of GR dimer is only twice higher than GR monomer. Then, expected ACF at channel *n* (G (green) or R (red)) of our experiment is

$$G_n(\tau) = \frac{N_{m,n}B_{m,n}{}^2 + N_{het,n}B_{het,n}{}^2 + N_{hom,n}B_{hom,n}{}^2}{\left(N_{m,n}B_{m,n} + N_{het,n}B_{het,n} + N_{hom,n}B_{hom,n}\right)^2} D_n(\tau), \tag{2}$$

where subscription *m*, *het*, and *hom* mean monomer (GFP-GR or mCh2-GR), hetero-dimer (GFP-GR and mCh2-GR), and homo-dimer (GFP-GR and GFP-GR, or mCh2-GR and mCh2-GR).

We define brightness of each species using a brightness of negative control of dimer formation such as GFP monomer $B_{nc,n}$ as follows:

$$B_{m,n} = B_{nc,n}, \tag{3}$$

$$B_{het,n} = B_{nc,n}, \tag{4}$$

$$B_{hom,n} = \beta_{B,n} B_{nc,n}, \tag{5}$$

where $\beta_{B,n}$ is the brightness ratio between negative control and homo-dimer measured by channel *n*. The number of each molecules in measurement volume are defined as

$$N_{m,n} = F_{m,n} N_{tot,n}, \tag{6}$$

$$N_{het,n} = F_{het,n} N_{tot,n}, \tag{7}$$

$$N_{hom,n} = F_{hom,n} N_{tot,n}, \tag{8}$$

$$F_{m,n} + F_{het,n} + F_{hom,n} = 1, \tag{9}$$

where F and N_{tot} are the number fraction of each species and total number of monomer, hetero-dimer, and homo-dimer, respectively. Using equations (3)-(9), equation (3) becomes

$$G_n(\tau) = \frac{F_{m,n}N_{tot,n}B_{nc,n}{}^2 + F_{het,n}N_{tot,n}B_{nc,n}{}^2 + F_{hom,n}N_{tot,n}\beta_{B,n}{}^2 B_{nc,n}{}^2}{\left(F_{m,n}N_{tot,n}B_{nc,n} + F_{het,n}N_{tot,n}B_{nc,n} + F_{hom,n}N_{tot,n}\beta_{B,n}B_{nc,n}\right)^2} D_n(\tau), \tag{10}$$

where $\beta_{N,n} = F_{hom,n} / F_{het,n}$ is the fraction/number ratio between homo- and hetero-dimer.

The apparent number of molecule obtained by non-linear least squares with two-componet diffusion model (Eq. (4) in the main manuscript) is

$$N_{app,n} = \frac{\left(F_{m,n} + F_{het,n} + \beta_{B,n}F_{hom,n}\right)^2}{F_{m,n} + F_{het,n} + \beta_{B,n}^2 F_{hom,n}} N_{tot,n}. \tag{11}$$

Using number fraction of all dimers $F_{n,d} = F_{n,het} + F_{n,hom}$, Eq. (11) can be simplified as follows:

$$N_{app,n} = \frac{\left(F_{m,n} + \beta_{1,n}F_{d,n}\right)^2}{F_{m,n} + \beta_{2,n}F_{d,n}} N_{tot,n}, \tag{12}$$

$$\beta_{1,n} = \frac{1 + \beta_{B,n}\beta_{N,n}}{1 + \beta_{N,n}}, \tag{13}$$

$$\beta_{2,n} = \frac{1 + \beta_{B,n}^2\beta_{N,n}}{1 + \beta_{N,n}}. \tag{14}$$

The average fluorescence intensity (count rate) observed at green channel is

$$\begin{aligned} \bar{I}_n &= \left(F_{m,n} + F_{het,n} + F_{hom,n}\beta_{B,n}\right) N_{tot,n}B_{nc,n} \\ &= \left(F_{m,n} + \beta_{1,n}F_{d,n}\right) N_{tot,n}B_{nc,n}. \end{aligned} \tag{15}$$

Using (11) and (12), the apparent molecular brightness (CPM) can be expressed as follows:

$$B_{app,n} = \frac{\bar{I}_n}{N_{apa,n}} = \frac{F_{m,n} + \beta_{2,n}F_{d,n}}{F_{m,n} + \beta_{1,n}F_{d,n}} B_{nc,n}. \tag{16}$$

Then, ratio of brightness between apparent brightness and the brightness of negative control can be obtained as

$$\alpha_{B,n} = \frac{B_{app,n}}{B_{nc,n}} = \frac{F_{m,n} + \beta_{2,n}F_{d,n}}{F_{m,n} + \beta_{1,n}F_{d,n}}. \tag{17}$$

From Eq. (17), fraction/number ratio between monomer and all dimers can be obtained as

$$\alpha_{N,n} = \frac{F_{m,n}}{F_{d,n}} = \frac{\beta_{2,n} - \beta_{1,n}\alpha_{B,n}}{\alpha_{B,n} - 1}. \tag{18}$$

Substituting Eq. (18) to Eq. (12),

$$N_{m,n} = \alpha_{N,n} \frac{\alpha_{N,n} + \beta_{2,n}}{\left(\alpha_{N,n} + \beta_{1,n}\right)^2} N_{app,n}, \tag{19}$$

$$N_{d,n} = F_{d,n} N_{tot,n} = \frac{\alpha_{N,n} + \beta_{2,n}}{\left(\alpha_{N,n} + \beta_{1,n}\right)^2} N_{app,n}. \tag{20}$$

where N_d is the number of all dimers (hetero- and homo-dimer). The concentrations of monomer C_m and dimer C_d contributing channel *n* are expressed as

$$C_{m,n} = \frac{N_{m,n}}{V_n} = [G] \ or \ [R], \tag{21}$$

$$C_{d,n} = \frac{N_{d,n}}{V_n} = [GG] + [RG] \ or \ [RR] + [RG], \tag{22}$$

where V_n is the effective confocal volume of channel *n*. G and R indicate the GFP-GR monomer and mCh2-GR mnomer, respectively. GG and RR are the homo-dimer of GFP-GR or mCh2-GR, respectively, and RG means the hetero-diemr of GFP-GR and mCh2-GR.

2. Fraction/number ratio between homo- and hetero-dimer ($\beta_{N,n}$)

To obtain the number of monomer and dimer using Eqs (19) and (20), the fraction/number ratio between homo- and hetero-dimer $\beta_{N,n}$ is needed. In the experiment, green labeled GR (GFP-GR) and red labeled GR (mCh2-GR) were used. Therefore, there were three kind of dimer, GG, RR, and RG. In this case, $\beta_{N,n}$ depends on the fraction/number ratio of mCh2-GR to GFP-GR, namely expression ratio of them. Using the same data of FCCS measurement, the expression ratio β_e can be obtained by following equation:

$$\beta_e = \frac{[R_0]}{[G_0]} = \frac{\bar{I}_R}{\bar{I}_G} \cdot \frac{B_{nc,G}}{B_{nc,R}} \cdot \frac{V_G}{V_R}, \tag{23}$$

where $[G_0]$ and $[R_0]$ mean concentrations of GFP-GR and mCh2-GR before dimer formation, respectively. V_n is a measurement volume of channel *n*. Using this expression ratio, the existing probability of each monomer in total monomers can be expressed as

$$P(G) = \frac{[G_0]}{[G_0] + [R_0]} = \frac{1}{1 + \beta_e}, \tag{24}$$

$$P(R) = \frac{[R_0]}{[G_0] + [R_0]} = \frac{\beta_e}{1 + \beta_e}. \tag{25}$$

Similarly, the existing probability of each kind of dimer in total dimers can be expressed as

$$P(GG) = P(G)^2, \tag{26}$$

$$P(RR) = P(R)^2, \tag{27}$$

$$P(RG) = 2P(G)P(R). \tag{28}$$

Then, the existing probability of homo-dimer can be obtained as

$$P(GG \cup RR) = P(G)^2 + P(R)^2. \tag{29}$$

Finally, the fraction/number ratio between homo- and hetero-dimer contributing to channel *n*, $\beta_{N,n}$ is given by

$$\beta_{N,G} = \frac{P(GG)}{P(RG)} = \frac{1}{2\beta_e}, \tag{30}$$

$$\beta_{N,R} = \frac{P(RR)}{P(RG)} = \frac{\beta_e}{2}. \tag{31}$$

3. K_d calculation

Dissociation constant of GR dimerization can be determined as

$$K_d = \frac{[M]^2}{[D]} = \frac{\left([\mathrm{G}]+[\mathrm{R}]\right)^2}{[GG]+[RR]+[RG]}, \tag{32}$$

where M and D indicate the GR monomer and dimer, respectively. Therefore, it is necessary to obtain the all number of monomer and dimer in the sample. From FCCS measurement of negative controls, $B_{nc,n}$ can be obtained. Subsequently, the fluorescence intensity $\overline{I}_n$, apparent number $N_{app,n}$, and apparent brightness $B_{app,n}$ can be obtained for each channel from measurement of sample. Using those parameters, relative brightness $\alpha_{B,n}$ and expression ratio β_e can be determined. The brightness ratio of homo-dimer to monomer $\beta_{B,n}$ should be also measured if it is possible. If it is impossible, the value is set to ideal value ($\beta_{B,n} = 2$). According to Eqs (13, 14, 18, 30, and 31), $\beta_{N,n}$, $\beta_{1,n}$, $\beta_{2,n}$, and $\alpha_{N,n}$ are calculated. Finally, we can obtain the concentration of monomer and dimer using Eqs (21 and 22). Substituting the concentrations to Eq. (32),

$$K_d = \frac{\left(C_{m,G} + C_{m,R}\right)^2}{C_{d,G} + C_{d,R} - C_{cross}}, \tag{33}$$

where C_{cross} indicate the concentration of hetero-dimer obtained by cross-correlation function of FCCS measurement.

Figure S1

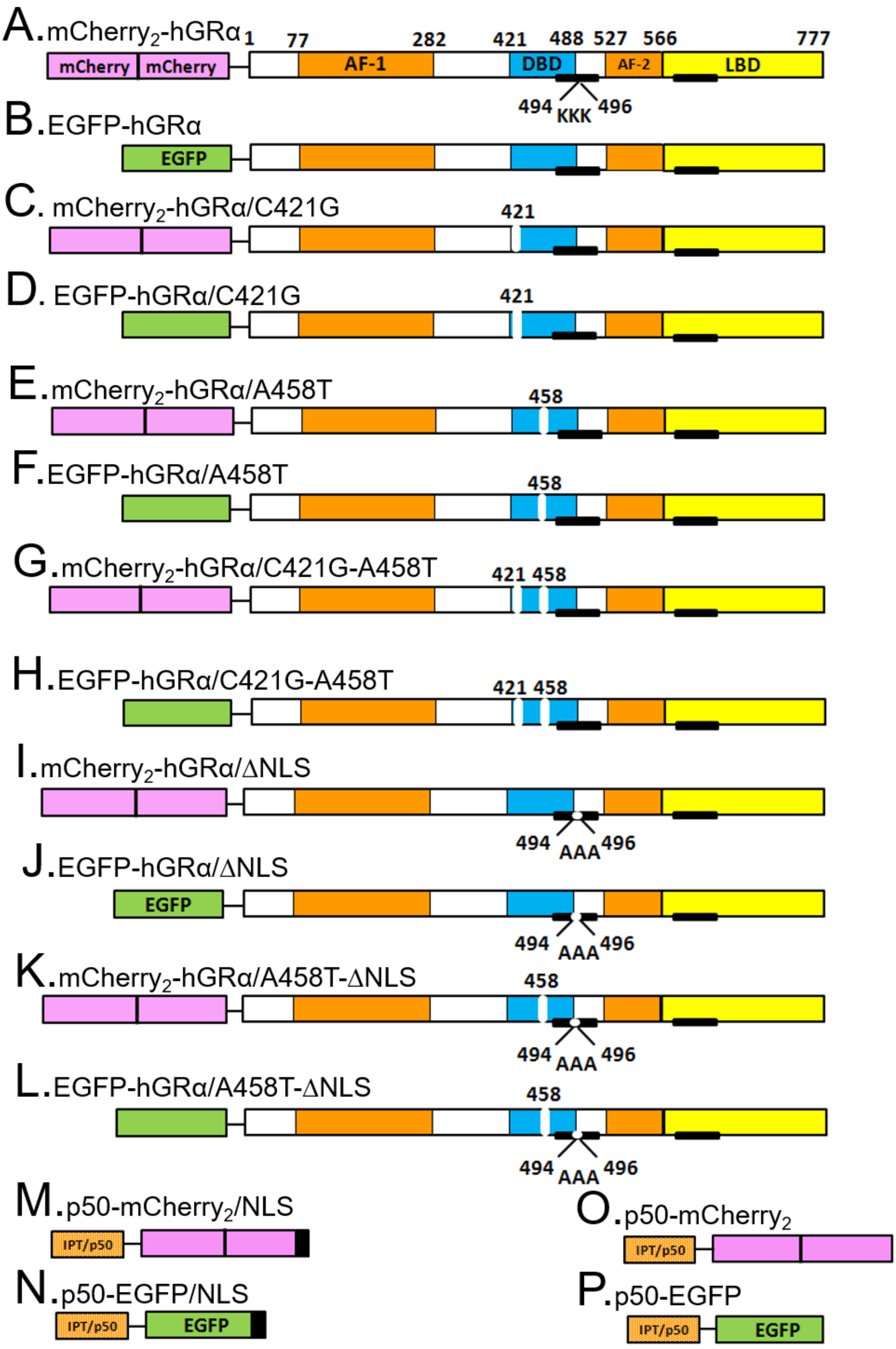
A. mCherry2-hGRα
1 77 282 421 488 527 566 777
mCherry mCherry AF-1 DBD AF-2 LBD
494 KKK 496
B. EGFP-hGRα
EGFP
C. mCherry2-hGRα/C421G
421
D. EGFP-hGRα/C421G
421
E. mCherry2-hGRα/A458T
458
F. EGFP-hGRα/A458T
458
G. mCherry2-hGRα/C421G-A458T
421 458
H. EGFP-hGRα/C421G-A458T
421 458
I. mCherry2-hGRα/ΔNLS
494 AAA 496
J. EGFP-hGRα/ΔNLS
EGFP
494 AAA 496
K. mCherry2-hGRα/A458T-ΔNLS
458
494 AAA 496
L. EGFP-hGRα/A458T-ΔNLS
458
494 AAA 496
M. p50-mCherry2/NLS
IPT/p50
N. p50-EGFP/NLS
IPT/p50 EGFP
O. p50-mCherry2
IPT/p50
P. p50-EGFP
IPT/p50 EGFP

Figure S1 Schematic representation of mCherry$_2$- and EGFP-fused constructs of WT and mutated hGRα

(A) and **(B):** A schematic diagram of wild-type hGRα showing the location of the N-terminal domain, activation function 1 domain (AF-1), DNA-binding domain (DBD), ligand-binding domain (LBD), and activation function 2 domain (AF-2). **(C)** and **(D):** In the C421G mutant, glycine was substituted for cysteine at position 421 (in the DNA-binding domain) to enable binding to GRE. **(E)** and **(F):** In the A458T mutant, threonine was substituted for alanine at position 458 (in the second zinc finger of the DNA-binding domain), resulting in a dimerization-deficient receptor. **(G)** and **(H):** The C421G-A458T mutant contains two point mutations. **(I)** and **(J):** In the ΔNLS mutant, three alanines were substituted for lysines from position 494 to position 496 (mutations K494A, K495A, and K496A), resulting in a nuclear-translocation-deficient receptor. **(K)** and **(L):** In the A458T-ΔNLS mutant, threonine was substituted for alanine at position 458 along with three lysine-to-alanine substitutions from position 494 to position 496, preventing both dimerization and translocation to the nucleus. The mutation sites are indicated by white spots. **(M)** and **(N):** The IPT domain of p50 fused with mCherry$_2$/NLS and EGFP/NLS, respectively. **(O)** and **(P):** The IPT domain of p50 fused with mCherry$_2$ and EGFP, respectively. The black region indicates the NLS sequence.

Figure S2

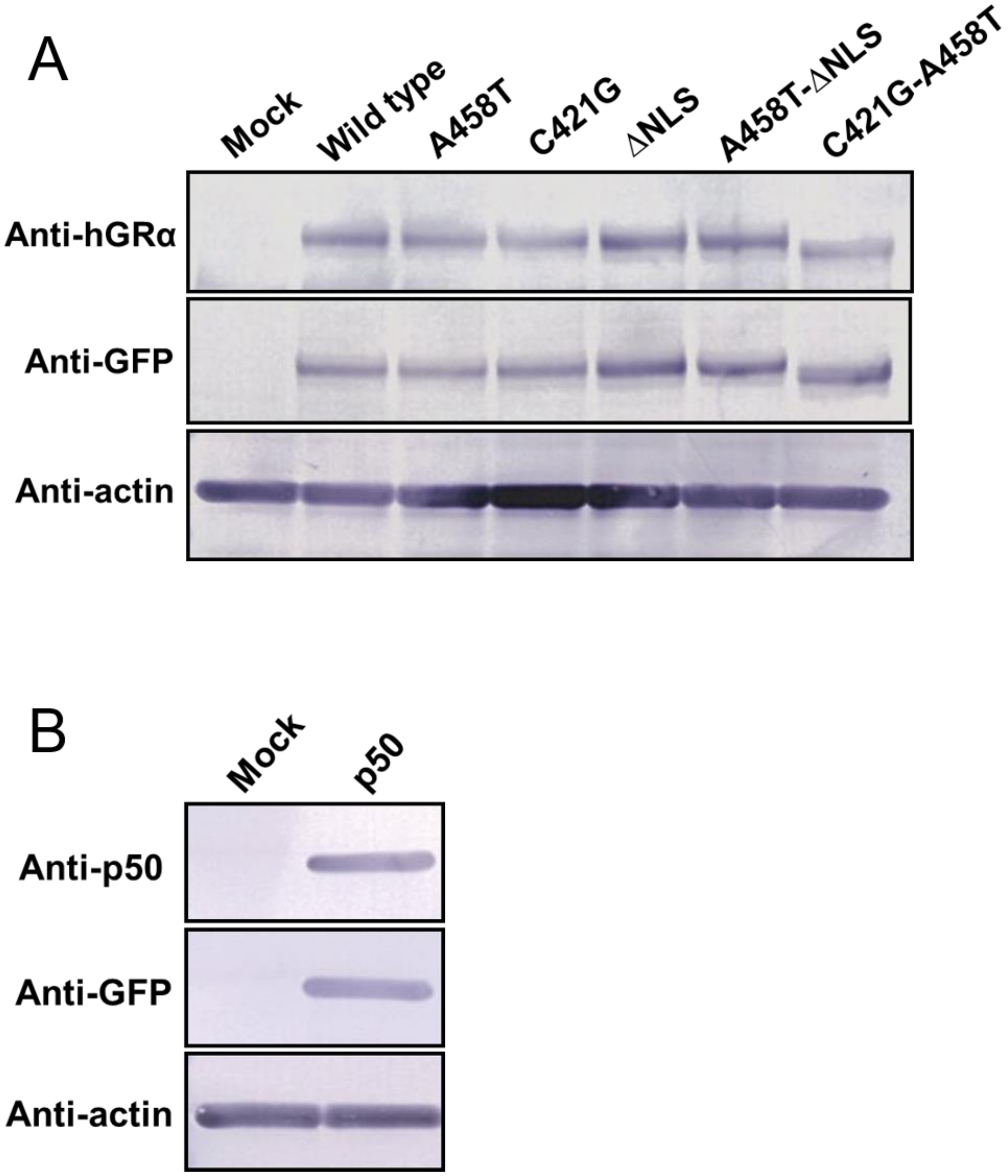

Figure S2 Endogenous hGRα and p50 are not expressed in U2OS cells

Western blot analysis of lysates of U2OS cells transfected with **(A)** EGFP-hGRα, EGFP-hGRα/A458T, EGFP-hGRα/C421G, EGFP-hGRα/ΔNLS, EGFP-hGRα/C421G-A458T, or EGFP-hGRα/A458T-ΔNLS; **(B)** p50-EGFP. Western blot analysis was performed using anti-hGRα, anti-p50, anti-GFP, and anti-actin antibodies. The whole images of western blots were shown in Fig. S15. All images were cropped at the cropped area (CA) in Fig. S15.

Figure S3

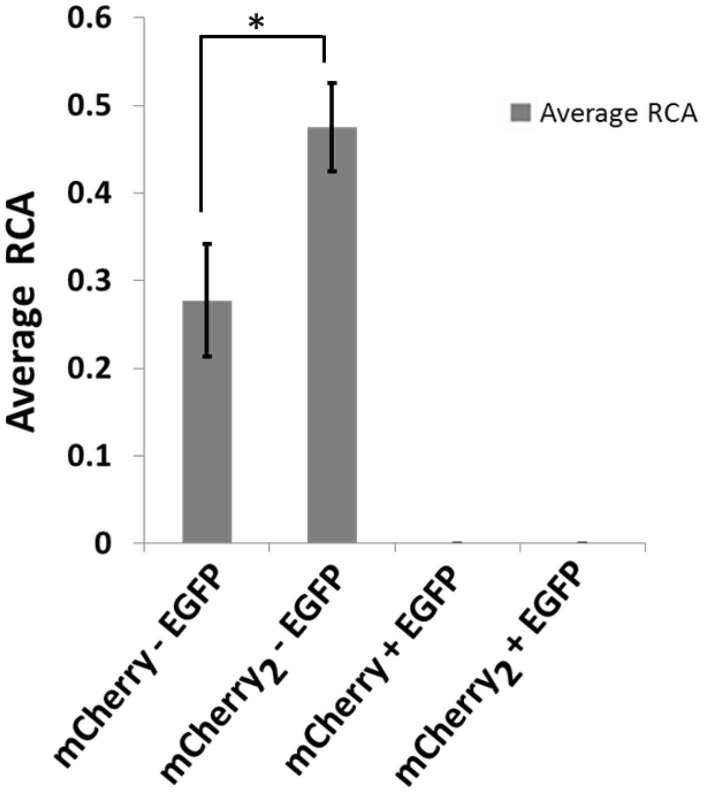

Figure S3 FCCS of mCherry-EGFP and $\mathbf{mCherry_2}$-EGFP

Relative cross-correlation, $[G_C(0) - 1]/[G_R(0) - 1]$, calculated from FCCS data corresponding to the fractions of the associated molecules (N_C/N_G). Here N_C is the average number of particles that have both green and red fluorescence in the excitation detection volume, and N_G is the number of the green fluorescent particles. Relative cross-amplitudes of mCherry-EGFP, $mCherry_2$-EGFP, mCherry and EGFP, $mCherry_2$ and EGFP. The data are presented as mean ± SD. Statistical analysis was based on the one-tailed *t* test ($*p < 0.01$).

Figure S4

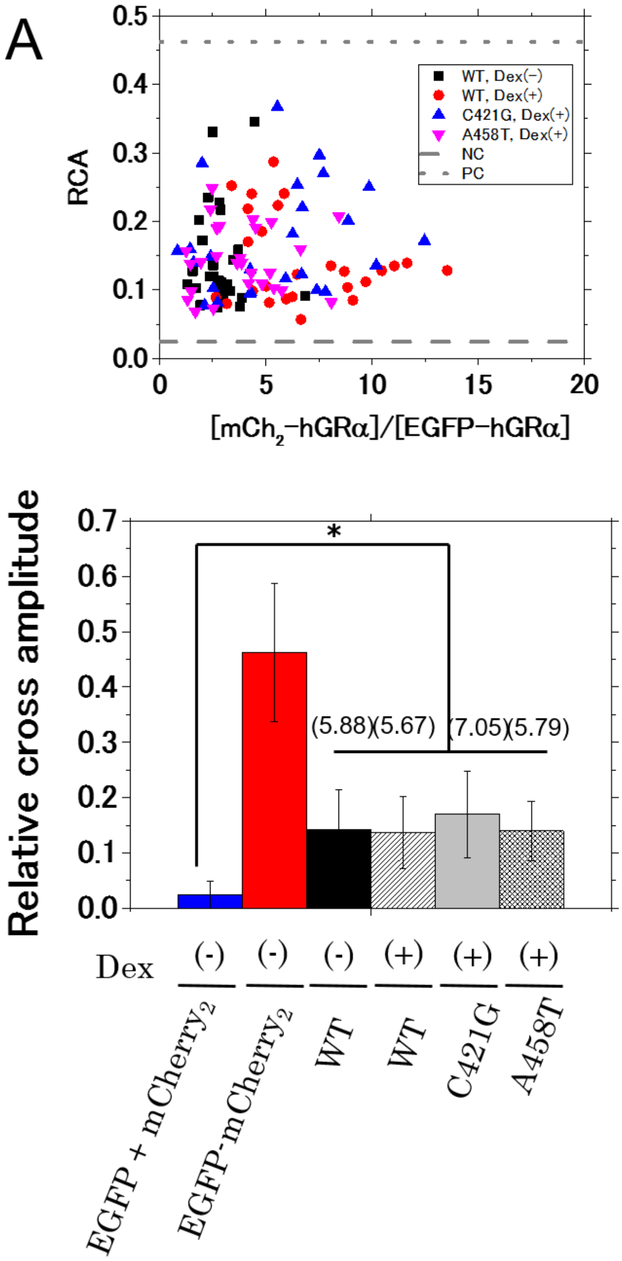

Figure S4 Comparison of relative cross-amplitudes (RCA) *in vitro* among the WT, mutants of hGRα, and coexpression of mCherry2 and EGFP

Relative cross-correlation amplitude (RCA), [$G_C(0)$ - 1]/[$G_R(0)$ - 1], was calculated from FCCS data. $G_C(0)$ and $G_R(0)$ denote the cross-correlation amplitude and autocorrelation amplitude in the red channel, respectively. **(A)**: Relative cross-amplitudes against concentration ratio between mCh2-hGRα and EGFP-hGRα. Black square: WT in the absence

of Dex, Red circle: WT in the presence of Dex, Blue up-triangle: C421G in the presence of Dex, Purple down-triangle: A458T in the presence of Dex, Dashed line: coexpression of $mCherry_2$ and EGFP as a negative control (concentration ratio: 4.6±1.1), Dotted line: tandem trimer of $mCherry_2$-EGFP as a positive control (concentration ratio: 1.6±0.4) **(B)**: Average RCA value for $mCherry_2$-hGRα and EGFP-hGRα in each construct. Blue filled bar:$mCherry_2$ and EGFP as a negative control (concentration ratio: 4.6±1.1), red filled bar: $mCherry_2$-EGFP as a positive control (concentration ratio: 1.6±0.4), black filled bar: the WT in the absence of Dex (concentration ratio: 2.9±1.1), hatched bar: the WT in the presence of Dex (concentration ratio: 6.5±2.9), gray filled bar: the C421G mutant in the presence of Dex (concentration ratio: 5.5±3.1), meshed bar: the A458T mutant in the presence of Dex (concentration ratio: 3.7±2.0). The data are presented as mean ± SD. Statistical analysis was based on the one-tailed *t* test (*$p < 0.05$). The bracketed number show fold changes of RCA values against that of negative control.

Figure S5

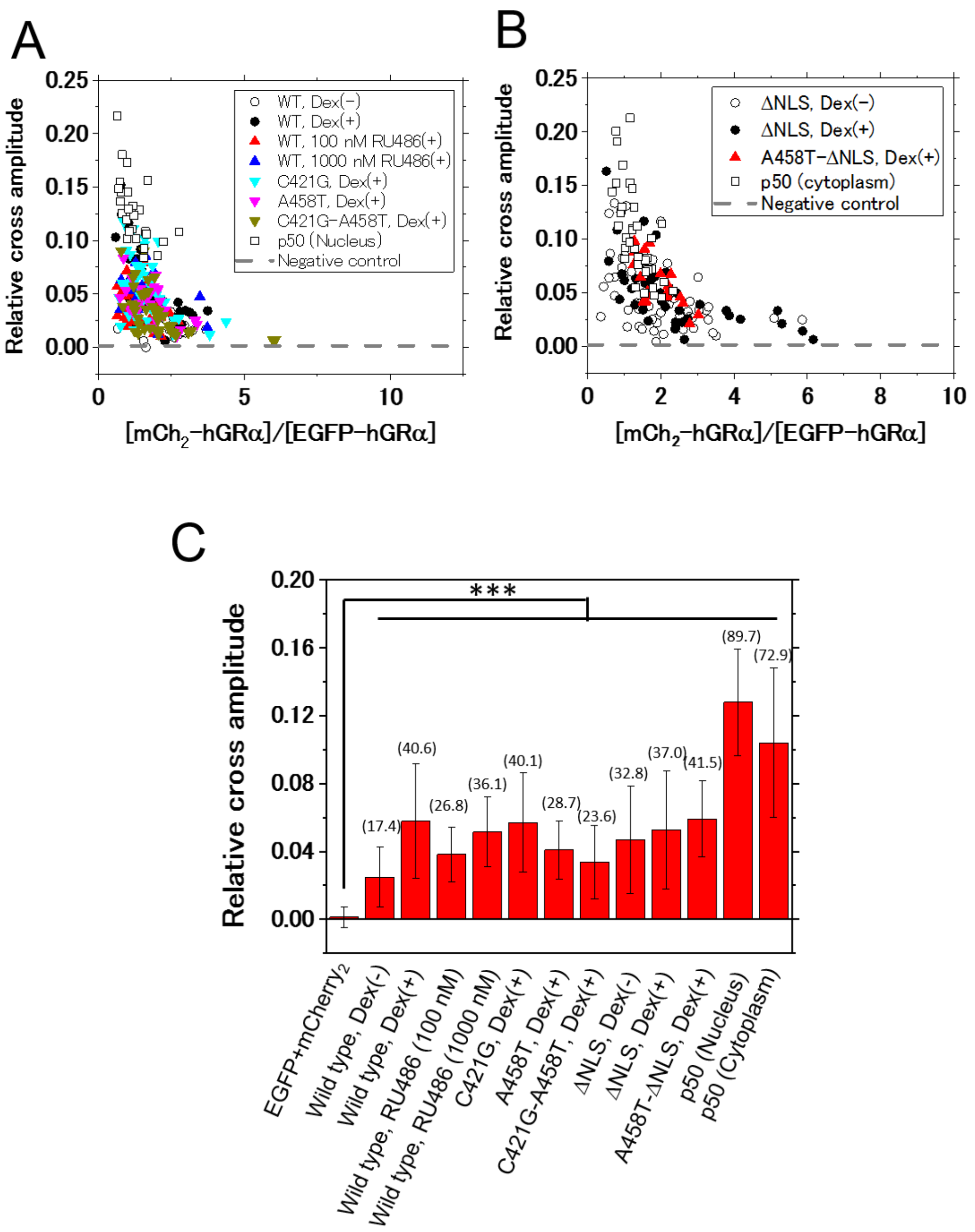

Figure S5 Comparison of relative cross amplitude (RCA) *in vivo* among hGRα and coexpression of mCherry2 and EGFP

Relative cross-correlation amplitude (RCA), [$G_C(0) - 1$]/[$G_R(0) - 1$], was calculated from FCCS data. $G_C(0)$ and $G_R(0)$ denote the cross-correlation amplitude and autocorrelation amplitude in the red channel, respectively. **(A)**: RCA of WT, C421G, A458T, C421G-A458T

and p50 against concentration ratio between mCh2- and EGFP-fused proteins. Black open circle: WT in the absence of Dex, Black filled circle: WT in the presence of Dex, Red up-triangle: WT in the presence of 100 nM RU486, Blue up-triangle: WT in the presence of 1000 nM RU486, Sky blue down-triangle: C421G in the presence of Dex, Purple down-triangle: A458T in the presence of Dex, Green down-triangle: C421G-A458T, Black open square: p50 (nucleus), Dashed line: coexpression of $mCherry_2$ and EGFP as a negative control (concentration ratio: 2.1±1.3). **(B):** RCA of ΔNLS mutants and p50 against concentration ratio between mCh2- and EGFP-fused proteins. Black open circle: ΔNLS in the absence of Dex, Black filled circle: ΔNLS in the presence of Dex, Red up-triangle: A458T-ΔNLS in the presence of Dex, Black open square: p50 (cytoplasm), Dashed line: coexpression of $mCherry_2$ and EGFP as a negative control (concentration ratio: 2.1±1.3). **(C):** Average RCA value for $mCherry_2$-hGRα and EGFP-hGRα in each construct.

EGFP+$mCherry_2$ (Concentration ratio: 2.1±1.3), Wild type in the absence of Dex (Concentration ratio: 2.1±0.7), Wild type in the presence of Dex (Concentration ratio: 1.8±0.8), Wild type in the presence of 100 nM RU486 (Concentration ratio: 1.4±0.5), Wild type in the presence of 1000 nM RU486 (Concentration ratio: 1.7±0.9), C421G in the presence of Dex (Concentration ratio: 1.6±0.8), A458T in the presence of Dex (Concentration ratio: 1.8±0.7), C421G-A458T in the presence of Dex (Concentration ratio: 2.0±1.1), ΔNLS in the absence of Dex (Concentration ratio: 2.0±1.1), ΔNLS in the presence Dex (Concentration ratio: 2.3±1.5), A458T-ΔNLS in the presence of Dex (Concentration ratio: 1.9 ±0.5), p50 (Nucleus) (Concentration ratio: 1.3±0.5), p50 (Cytoplasm) (Concentration ratio: 1.4±0.4). The data are presented as mean ± SD. Statistical analysis was based on the one-tailed *t* test (*** $p < 0.001$). The bracketed number show fold changes of RCA values against that of negative control.

Figure S6

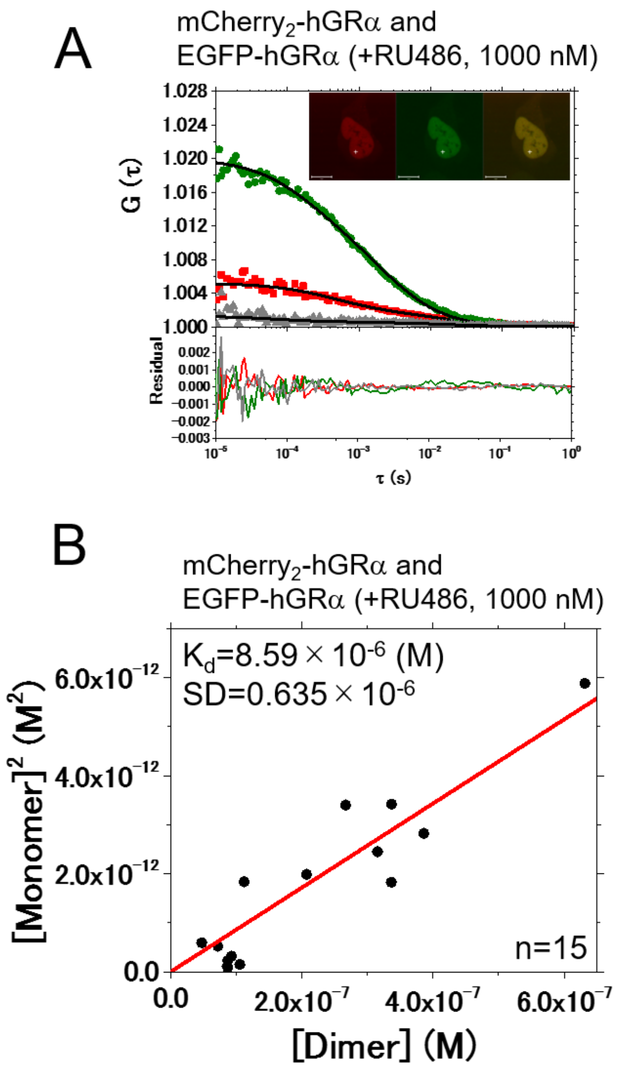

Figure S6. The homodimerization of hGRα by high concentration of RU486 analyzed by FCCS

Typical auto- and cross-correlation curves obtained from U2OS cells coexpressing the pairs of chimeric fusion proteins before and after addition of the ligand. The filled green diamonds, red squares, and gray triangles denote autocorrelation of the green channel [$G_G(\tau)$], autocorrelation of the red channel [$G_R(\tau)$], and the cross-correlation curve [$G_C(\tau)$], respectively, with their fits (solid black lines) and residuals. The insets show LSM images of U2OS cells coexpressing the pairs of chimeric fusion proteins. Measurement positions of FCCS are indicated by the white crosshairs. The scale bars are 10 μm. **(A)** FCCS was

performed in U2OS cells coexpressing mCherry$_2$-hGRα and EGFP-hGRα in the nucleus 20 min after addition of 1000 nM RU486. **(B)** The K_d plot represents the square of the concentrations of the monomeric hGRα versus the concentration of the dimer of hGRα after addition of RU486. The solid line shows the linear fit.

Figure S7

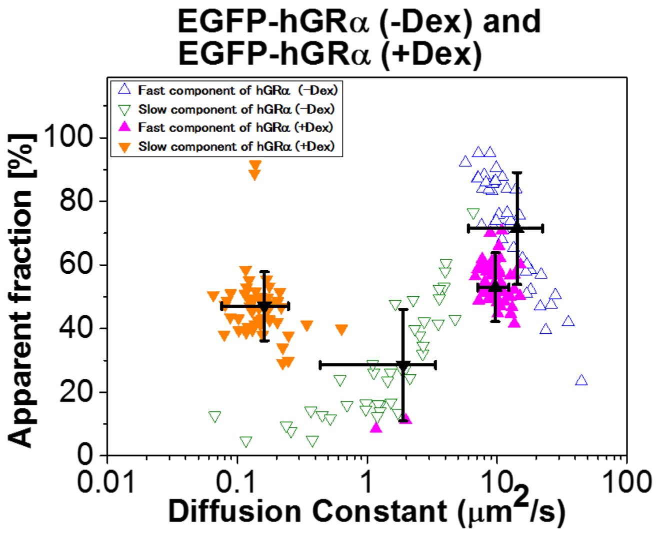

Figure S7 A scatter plot of diffusion constants of WT GRα in the presence and absence of Dex

Comparative analysis of diffusion constants of EGFP-fused WT hGRα before and after addition of Dex. The scatter plots represent the diffusion constants versus their apparent fractions from FCCS data. Black symbols indicate the average diffusion constants of the fast and slow components. Error bars are SD. Open triangles: WT (without Dex) in the cytoplasm, filled triangles: WT (+Dex) in the nucleus. The triangles and inverted triangles represent the fast and slow components, respectively.

Figure S8

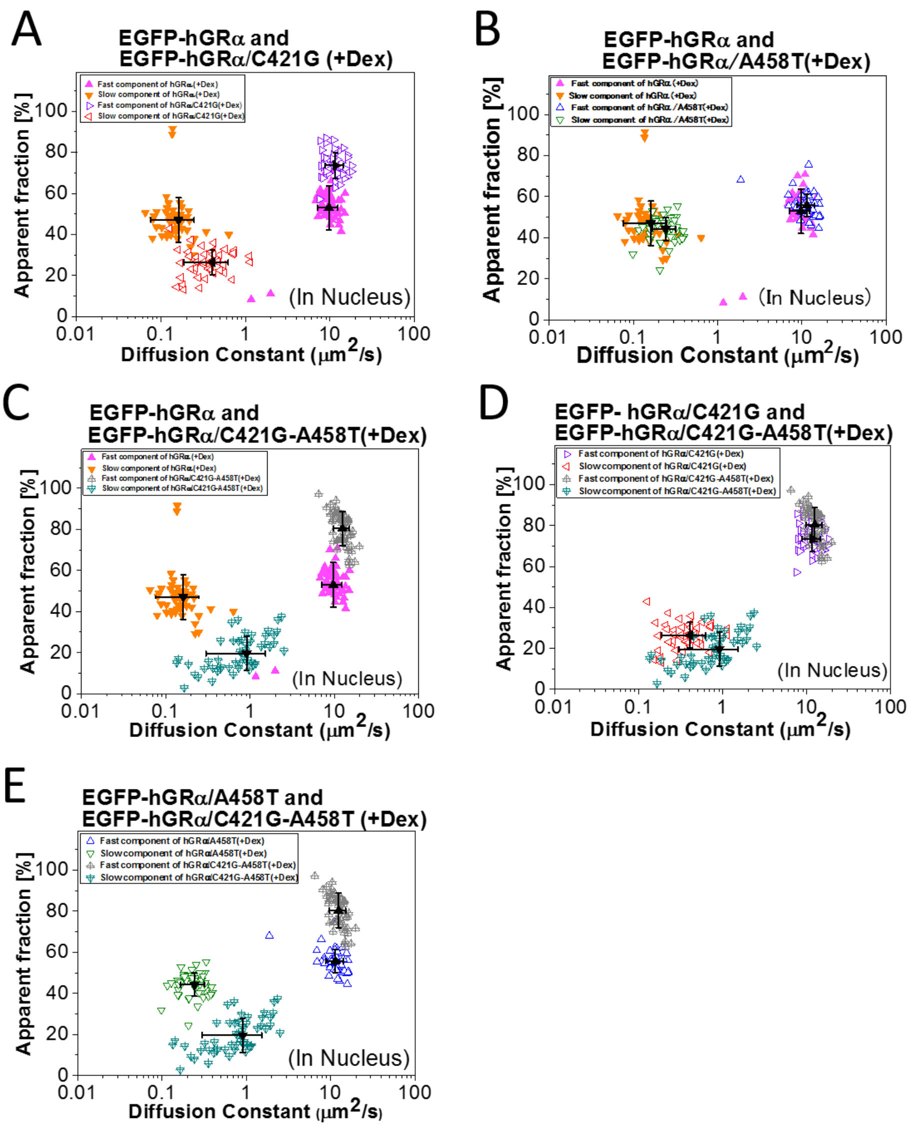

Figure S8 Scatter plots of diffusion constants of the WT and mutants of GRα

Comparative analysis of diffusion constants of EGFP-fused WT hGRα and mutants after addition of Dex. The scatter plots represent the diffusion constants versus their apparent fractions from FCCS data. Black symbols indicate the average diffusion constants of the fast and slow components. Error bars are SD. Filled triangles: WT, open triangles: mutants (C421G or A458T). Triangles containing a small cross: double mutant (C421G-A458T). The triangles pointing up or to the right represent the fast component, and the inverted triangles and those pointing to the left represent the slow component.

Figure S9

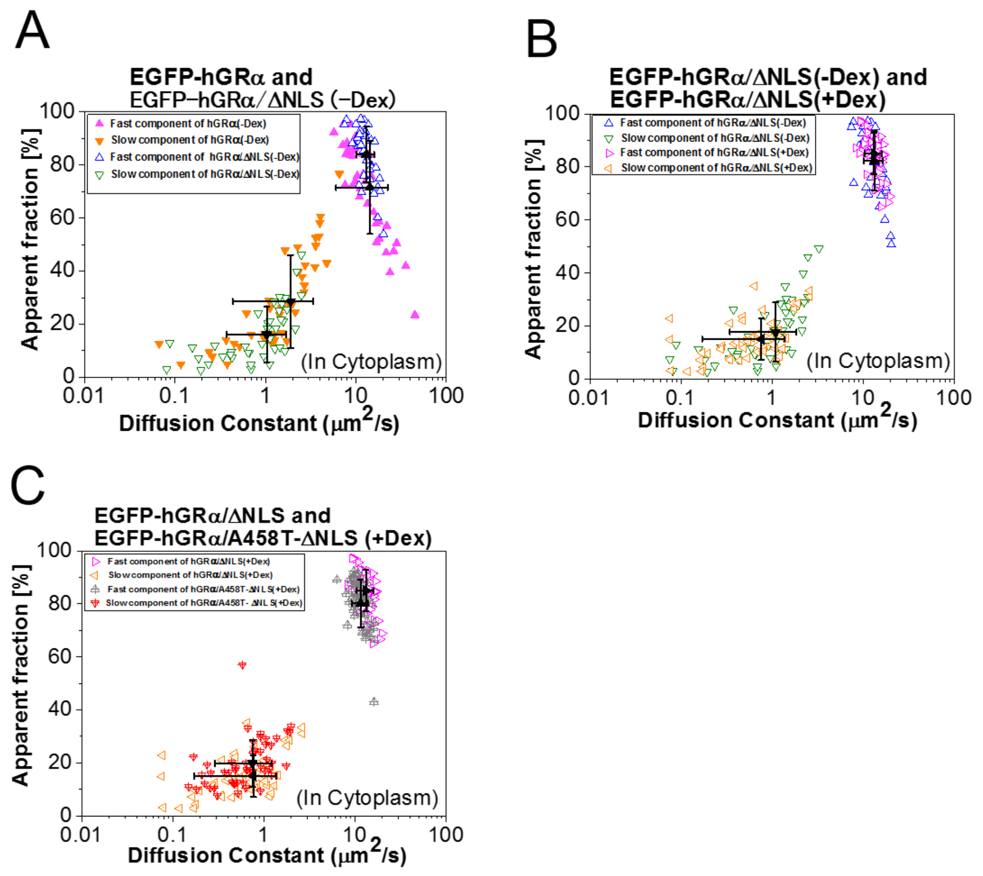

Figure S9 Scatter plots of diffusion constants of the WT and ΔNLS mutants of GRα

Comparative analysis of diffusion constants of EGFP-fused WT hGRα and ΔNLS mutants before and after addition of Dex. The scatter plots represent the diffusion constants versus their apparent fractions from FCCS data. Black symbols indicate the average diffusion constants of the fast and slow components. Error bars are SD. Filled triangles: WT, open triangles and those with dots in the center represent mutant (ΔNLS) before and after addition of Dex, respectively. Triangles containing a small cross: double mutant (A458T-ΔNLS). The upward- or right-pointing triangles represent the fast component, whereas the inverted and left-pointing triangles represent the slow component.

Figure S10

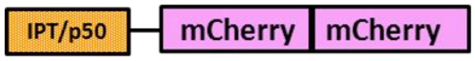

ATGAATGCATCCAACTTGAAAATTGTAAGAATGGACAGGACAGCTGGATGTGTGACTGGAGGGGAG

GAAATTTATCTTCTTTGTGACAAAGTTCAGAAAGATGACATCCAGATTCGATTTTATGAAGAGGAAG

AAAATGGTGGAGTCTGGGAAGGATTTGGAGATTTTTCCCCCACAGATGTTCATAGACAATTTGCCAT

TGTCTTCAAAACTCCAAAGTATAAAGATATTAATATTACAAAACCAGCCTCTGTGTTTGTCCAGCTTC

GGAGGAAATCTGACTTGGAAACTAGTGAACCAAAACCTTTCCTCTACTATCCTGGA gcggccgccaattccg

ctgacggcggcggaggatcgggtggtagtggtggttcaggaggaggatcgacccaaggaccggtcgccacc**ATG**

Start codon of
mCherry$_2$

Figure S10 Sequence of the subunit of the NF-κB protein

Sequence of the IPT domain of p50 fused with mCherry$_2$. Underlining indicates the start codon of mCherry$_2$.

Figure S11

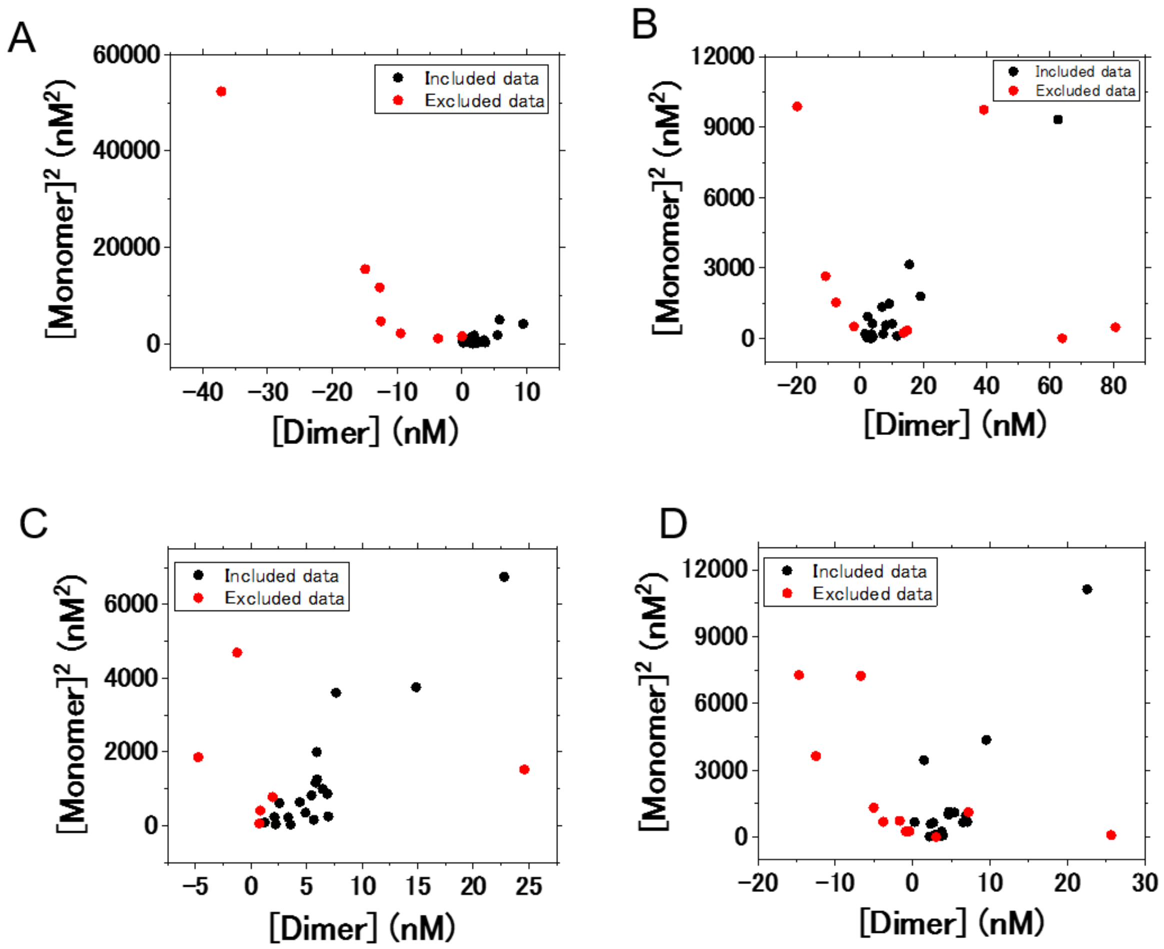

Figure S11 Whole data plots of squared monomer concentration against dimer concentration *in vitro* experiments

The monomer and dimer concentrations were calculated by supplemental method. The graphs show the square of monomer concentration, ([G]+[R])^2 against dimer concentration, [GG]+[RR]+[RG]. Several data points in which concentrations of each fluorescently-tagged monomer ([G] or [R]) or homo-color dimer ([GG] or [RR]) show minus values were excluded from the K_d determination. It is noted that the square of monomer concentration, ([G]+[R])^2 is positive values in which [G] or [R] show minus values, and that dimer concentration, [GG]+[RR]+[RG] may be positive values, even if [GG] or [RR] show minus values. **(A):** Wild type in the absence of Dex (Excluded data number/Whole data number:

7/28), **(B):** Wild type in the presence of Dex (Excluded data number/Whole data number: 9/27), **(C):** C421G in the presence of Dex (Excluded data number/Whole data number: 6/25), **(D):** A458T in the presence of Dex (Excluded data number/Whole data number: 11/28). Black filled circle: Included data, Red filled circle: Excluded data.

Figure S12

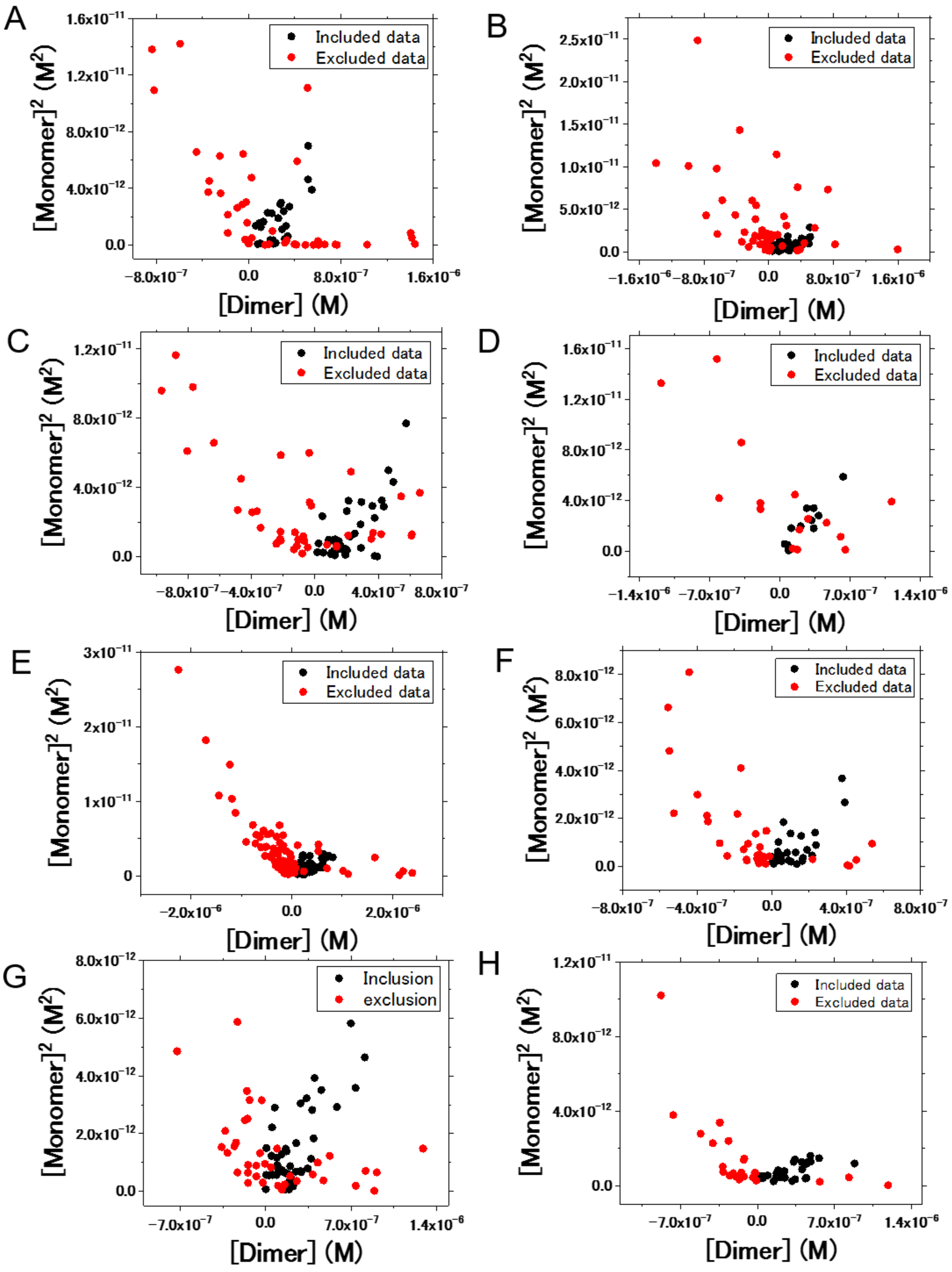
A
B
C
D
E
F
G
H
[Monomer]^2 (M^2)
[Dimer] (M)
Included data
Excluded data
Inclusion
exclusion
1.6x10^{-11}
1.2x10^{-11}
8.0x10^{-12}
4.0x10^{-12}
0.0
−8.0x10^{-7}
8.0x10^{-7}
1.6x10^{-6}
2.5x10^{-11}
2.0x10^{-11}
1.5x10^{-11}
1.0x10^{-11}
5.0x10^{-12}
−1.6x10^{-6}
−4.0x10^{-7}
4.0x10^{-7}
−1.4x10^{-6}
−7.0x10^{-7}
7.0x10^{-7}
1.4x10^{-6}
3x10^{-11}
2x10^{-11}
1x10^{-11}
0
−2.0x10^{-6}
2.0x10^{-6}
6.0x10^{-12}
2.0x10^{-12}

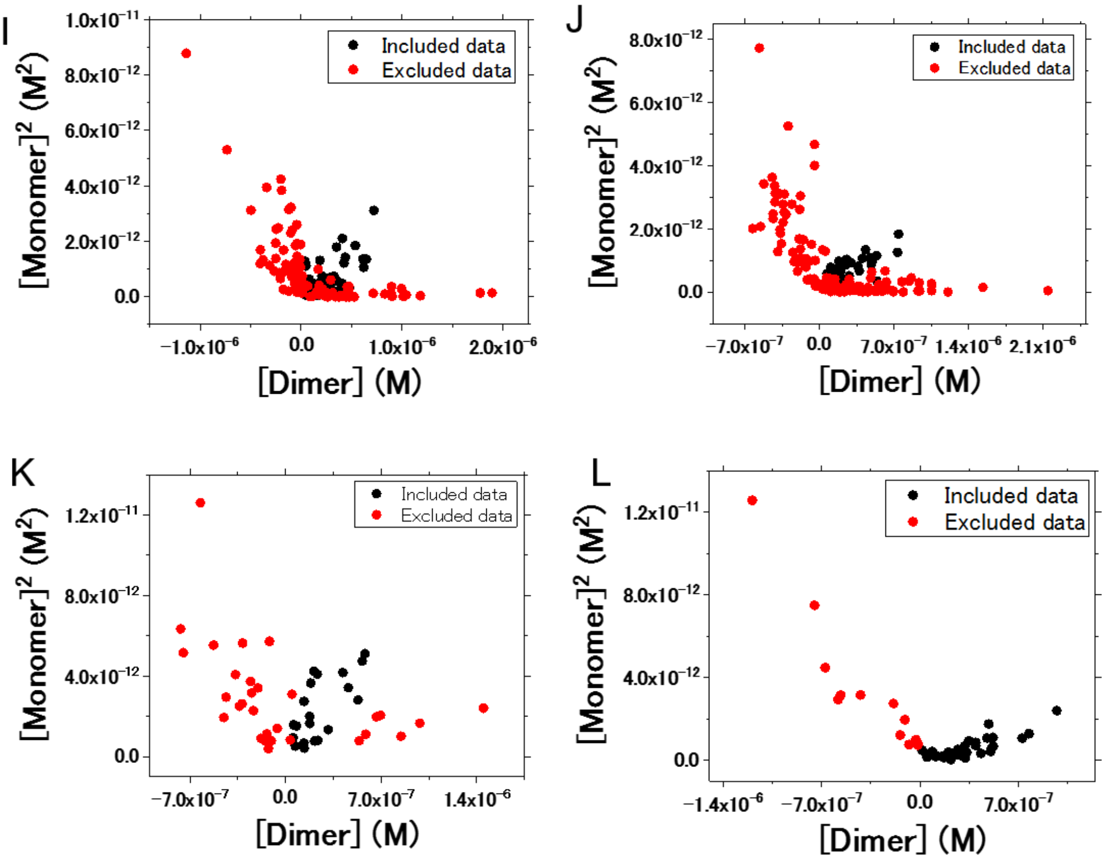

Figure S12 Whole data plots of squared monomer concentration against dimer concentration *in vivo* experiments

The monomer and dimer concentrations were calculated by supplemental method. The graphs show the square of monomer concentration, $([G]+[R])^2$ against dimer concentration, [GG]+[RR]+[RG]. Several data points in which concentrations of each fluorescently-tagged monomer ([G] or [R]) or homo-color dimer ([GG] or [RR]) show minus values were excluded from the K_d determination. It is noted that the square of monomer concentration, $([G]+[R])^2$ is positive values in which [G] or [R] show minus values, and that dimer concentration, [GG]+[RR]+[RG] may be positive values, even if [GG] or [RR] show minus values. **(A):** Wild type in the absence of Dex (Excluded data number/Whole data number: 41/65), **(B):** Wild type in the presence of Dex (Excluded data number/Whole data number: 44/80), **(C):** Wild type in the presence of 100 nM RU486 (Excluded data number/Whole data

number: 40/78), **(D):** Wild type in the presence of 1000 nM RU486 (Excluded data number/Whole data number: 15/30), **(E):** C421G in the presence of Dex (Excluded data number/Whole data number: 64/103), **(F):** A458T in the presence of Dex (Excluded data number/Whole data number: 37/63), **(G):** C421G-A458T in the presence of Dex (Excluded data number/Whole data number: 37/79), **(H):** p50 in the nucleus (Excluded data number/Whole data number: 24/50), **(I):** ΔNLS in the absence of Dex (Excluded data number/Whole data number: 93/151), **(J):** ΔNLS in the presence of Dex (Excluded data number/Whole data number: 120/159), **(K):** A458T-ΔNLS in the presence of Dex (Excluded data number/Whole data number: 30/50), **(J):** p50 in the cytoplasm (Excluded data number/Whole data number: 13/50). Black filled circle: Included data, Red filled circle: Excluded data.

Figure S13

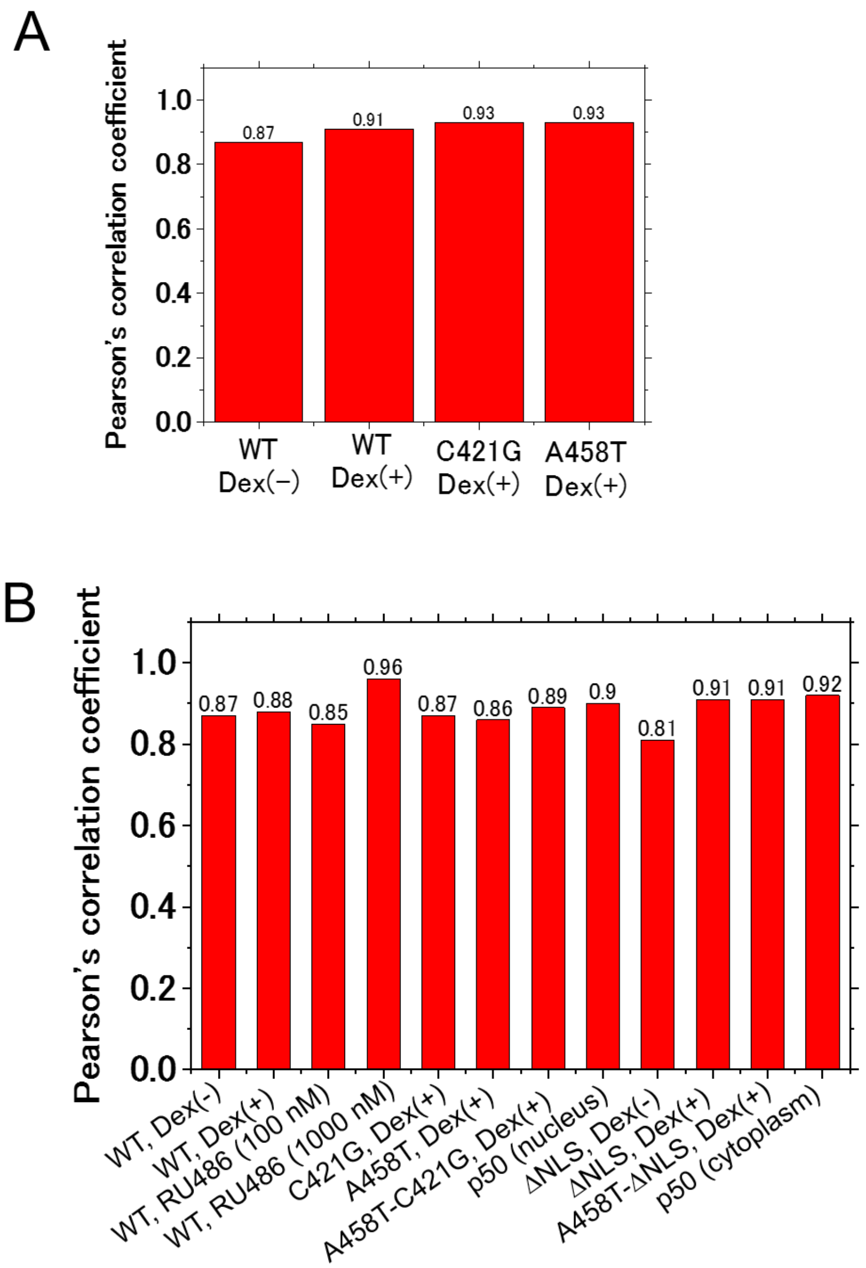

Figure S13 Pearson's correlation coefficient of fitting analysis *in vitro* and *in vivo*

Pearson's correlation coefficient was determined by linear least-square fitting to the data **(A)** *in vitro* and **(B)** *in vivo* for the K_d determination. All mutant show the higher value than 0.6, suggesting strong relationship between the square concentration of monomeric hGRα and the concentration of dimeric hGRα.

Figure S14

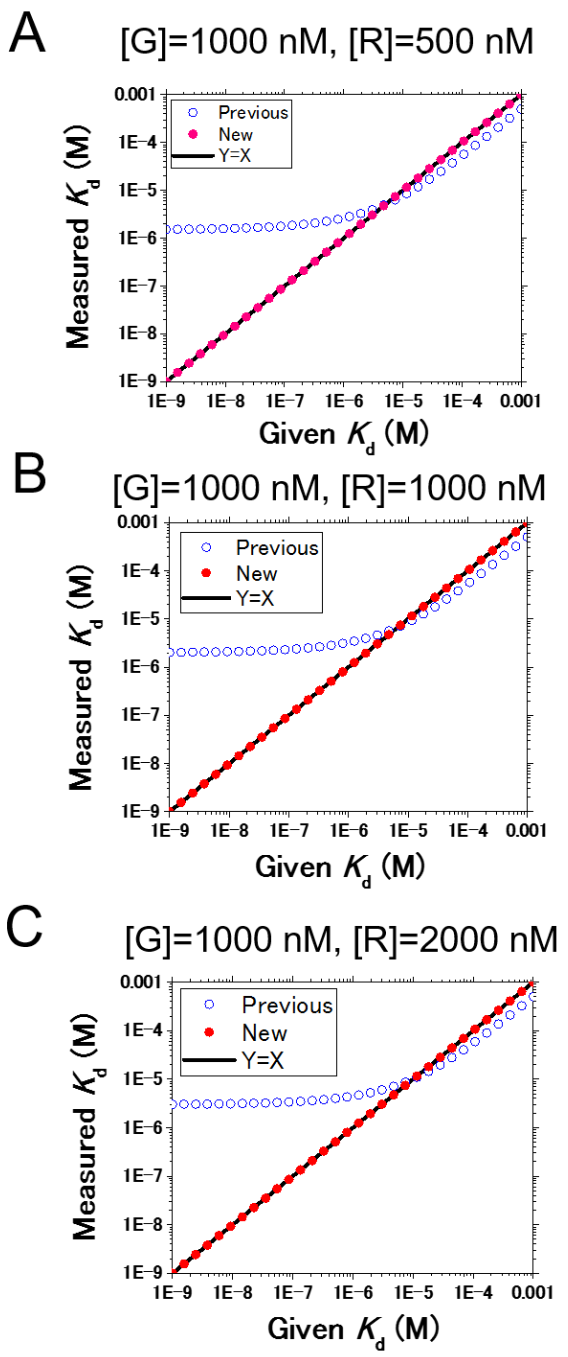

Figure S14 Simulation analysis of the measured K_d value against the given K_d value

Two K_d calculation methods without or with the consideration of homo-color dimer were simulated to determine the K_d calculation method from FCCS measurement data. The simulations were performed at the three conditions of expression level for EGFP-hGRα and mCherry$_2$-hGRα: **(A)** 1000 nM EGFP-hGRα (G), 500 nM mCherry$_2$-hGRα (R); **(B)** 1000

nM EGFP-hGRα (G), 1000 nM mCherry$_2$-hGRα (R); **(C)** 1000 nM EGFP-hGRα (G), 2000 nM mCherry$_2$-hGRα (R). The given K_d value cannot be re-obtained using the calculation method without the consideration of homo-color dimer (Blue open circle), in contrast the calculation method with the consideration of homo-color dimer determines the same measured K_d value with the given K_d value (Red filled circle). The previous K_d calculation equation without the consideration of homo-color dimer is shown, as below

$$(\text{previous } K_d) = \frac{([\mathrm{G_T}] - [\mathrm{RG}]) \times ([\mathrm{R_T}] - [\mathrm{RG}])}{[\mathrm{RG}]} \qquad (34)$$

where, $[G_T]$ and $[R_T]$ denote the concentration of EGFP-hGRα and mCh$_2$-hGRα from autocorrelation amplitude, and [RG] is the concentration of hetero-color dimer between EGFP-hGRα and mCh$_2$-hGRα from cross-correlation amplitude.

The new K_d calculation equation with the consideration of homo-color dimer is shown in the equation (32), as following

$$(\text{new } K_d) = \frac{([\mathrm{G}] + [\mathrm{R}])^2}{[\mathrm{GG}] + [\mathrm{RR}] + [\mathrm{RG}]} \qquad (35)$$

where, [G] and [R] denote the concentration of monomeric EGFP-hGRα and mCh$_2$-hGRα, and [GG] and [RR] show the homo-color dimer of EGFP-hGRα and mCh$_2$-hGRα, respectively.

Figure S15

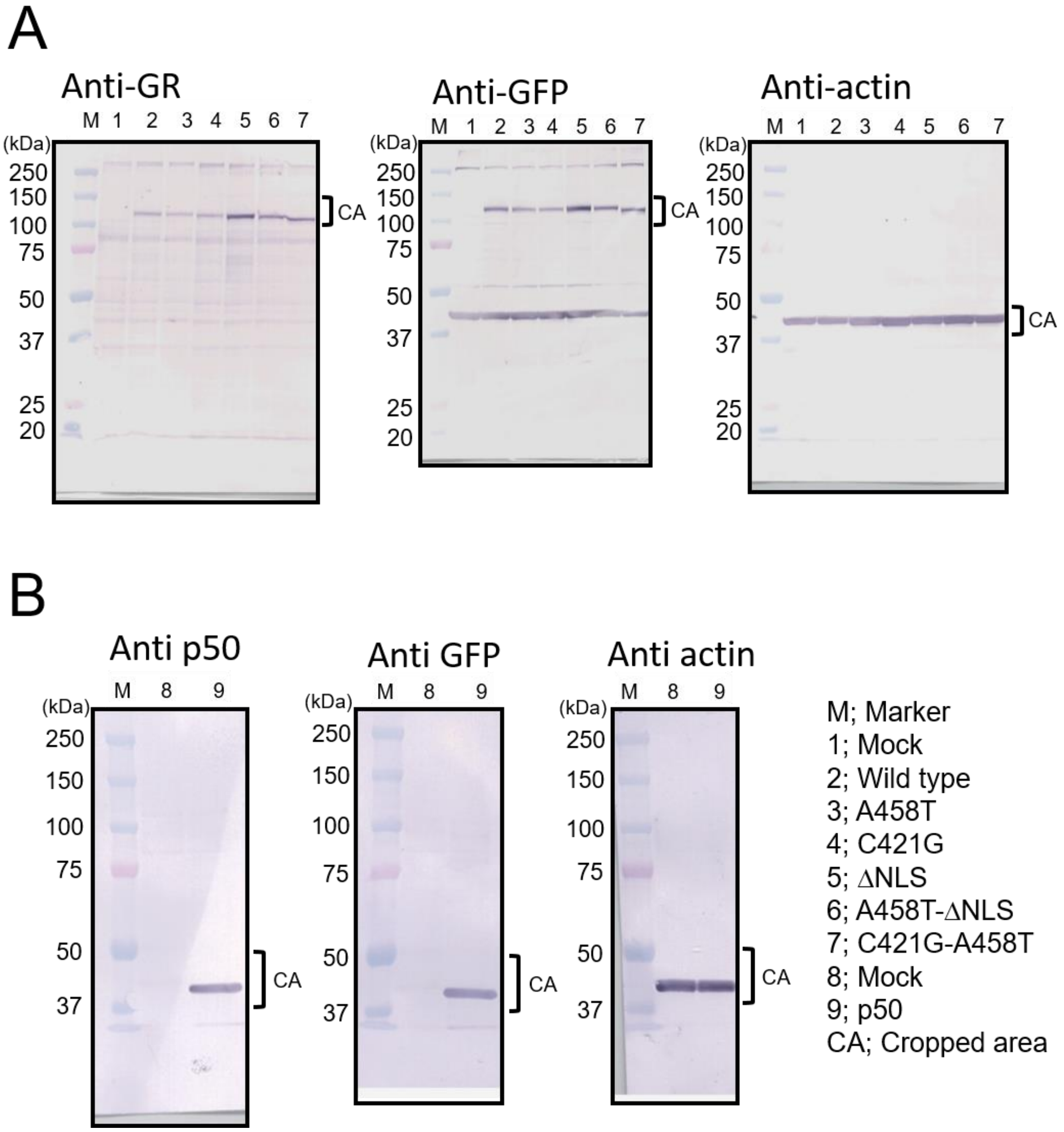

Figure S15 Whole images of western blots in Fig. S2

Whole images of western blots were shown. Fig. S2 shows the images cropped at the cropped area (CA). **(A)** Western blot of EGFP-hGRα, EGFP-hGRα/A458T, EGFP-hGRα/C421G, EGFP-hGRα/ΔNLS, EGFP-hGRα/C421G-A458T, or EGFP-hGRα/A458T-ΔNLS. **(B)** Western blot of p50-EGFP
